# Supplementary material for: Unraveling the neurophysiological correlates of phase-specific enhancement of motor memory consolidation via slow-wave closed-loop targeted memory reactivation
Source: Nat Commun. 2025 Mar 18;16:2644. doi: 10.1038/s41467-025-57602-2 (PMC11920436; doi:10.1038/s41467-025-57602-2)
Supplement: Supplementary file 1 — Supplementary Information [file 41467_2025_57602_MOESM1_ESM.pdf]

Supplemental information of the manuscript entitled

Unraveling the Neurophysiological Correlates of Phase-Specific Enhancement of Motor Memory Consolidation via Slow-Wave Closed-Loop Targeted Memory Reactivation

Authors

Judith Nicolas, Bradley R. King, David Levesque, Latifa Lazzouni, Gaëlle Leroux, David Wang, Nir Grossman, Stephan P. Swinnen, Julien Doyon, Julie Carrier, Geneviève Albouy

Table of content

1. **Cue-locked electrophysiological analyses** 3
Figure S1: Results of the cue-locked electrophysiological analyses. 5
2. **Trough-locked electrophysiological analyses on all channels** 7
Figure S2 (previous page): Channel-level EEG data. 9
3. **Sleep event detections** 9
Figure S3: Offline detected sleep events. 9
4. **Main effect of practice (across sequences) and its linear modulation by performance** 10
Figure S4: Brain activity elicited by the practice of the motor sequence learning task. 10
Table S1: Functional imaging results of the main effect of motor sequence learning. 10
5. **Activation-based results reported in the main text** 12
Table S2: Functional imaging results for the activation-based analyses. 12
6. **Connectivity results reported in the main text** 17
Table S3: Functional imaging results for the connectivity analyses. 17
7. **Brain-behavior regression analyses using between-condition activation maps** 26
Figure S5: Overlay of the within-condition and between-condition contrasts regression analyses.. 27
8. **Supplementary regressions analyses between connectivity and performance/sleep measures** 28
Figure S6: Regressions between connectivity, behavior and EEG metrics. u 28
9. **List of the deviations from the pre-registration** 29
Table S4. List of the deviations from the pre-registered analyses followed by their justification.... 29
10. **Participant characteristics** 31
Table S5. Participants' characteristics, sleep and vigilance 31
11. **Negative control analyses** 33
Impact of the number of cues on offline changes in performance 33
Equivalent number of discarded trials in the different sessions and conditions 33
Table S6: Mean percent of discarded trials in the behavioral analysis 33
Equivalent vigilance during each behavioral session 33
Equivalent baseline performance between the three movement sequences 33
Figure S7: Behavioral results per sequence. 34
Equivalent baseline performance between the three conditions 34
Equivalent impact of the three different sounds 35

Figure S8: Offline changes in performance speed as a function of the sound................................ 35
Motor execution performance 35
12. Confirmatory analysis on accuracy 36
Figure S9: Behavioral results on accuracy data... 36
13. All NREM stimulation analysis.. 37
Figure S10: Electrophysiological results of the analyses including the NREM2-3 sleep stimulations. 38
14. Seed regions for connectivity analyses 39
Figure S11: Seed regions for the connectivity analyses.. 39
15. Functional role of the brain regions selected a priori for the MRI analyses.................................... 40
16. Coordinates for small volume corrections 41
Table S7: Coordinates of areas of interest used for spherical small volume corrections.................. 41
Table S8: Description of the published works used for spherical small volume corrections. 42
References 43

1. Cue-locked electrophysiological analyses

ERP analyses: The results of the cue-locked ERP analyses are presented in Figure S1a below and show that up-stimulation, as compared to up-sham, did not elicit an increase in response amplitude *during cue presentation* but was followed by a deflection of greater amplitude (significant spatio-temporal clusters are shown with the horizontal magenta lines in Figure S1a). This is partially in line with earlier observations as Ngo and Staresina [9] reported a similar deeper deflection as observed in the current study after cue offset. However, they also reported a prolonged up-state during cue presentation that we did not observe in the current study. The discrepancy in results during cue presentation might be related to the difference in sound duration between the two studies. The current study used a much shorter auditory cue (duration 100ms as compared to 500ms in Ngo and colleagues) that might have induced less pronounced effect *during* sound exposure. Interestingly, we report greater amplitude modulation for the up-stimulated SO (as compared to sham) **before cue-onset** while no such effect is reported in Ngo and Staresina [9]. Although it is difficult to compare results between studies as no statistical analyses were performed prior cue-onset in [9], this earlier work shows less pronounced modulations of SO amplitude prior to cue onset. As discussed above, we suggest that this discrepancy is attributed to the difference in stimulation designs. Specifically, in this earlier research, four different stimulation conditions were used (i.e., up-associated, up-unassociated, down-associated and down-unassociated) and the corresponding sounds were presented randomly with an 8 sec. pause between each stimulation. In contrast, stimulation in our research was performed using a block design that allowed the stimulation of consecutive SOs [90.83 % [95CI: 87.13 – 94.53] of the auditory cues were sent at a frequency that corresponds to the SO frequency range (i.e., 0.1 to 4.5 Hz)] in a sustained manner during 3-min epoch for each stimulation condition. The cue-locked ERP results suggest that our stimulation design particularly favored the entrainment of ongoing sleep oscillations. Interestingly, while the 500-ms acoustic stimulation in [9] consistently elicited a second SO cycle after cue-offset, no such effect was observed in our study with a shorter sound duration (100ms). In contrast, entrainment in our study is reflected by the pre-cue modulations discussed above.

The comparison between down-stimulation and down-sham conditions (Figure S1a, significant spatio-temporal clusters are shown with the horizontal blue lines) indicated that stimulation resulted in a decrease in SO trough amplitude during and up to 400ms after cue exposure. Similar to the up condition, significant amplitude modulation was observed pre-cue whereby SO amplitude was greater for down-stimulation as compared to sham which might also reflect oscillatory entrainment as discussed above. Similar as above, while the 500-ms down acoustic stimulation in [9] consistently elicited a second SO cycle after cue-offset, no such effect was observed in our study in which entrainment in the down condition was also reflected by pre-cue amplitude modulations.

Finally, the direct comparison between up- and down-stimulation conditions (significant spatio-temporal clusters are shown with the horizontal black lines in Figure S1a) showed that the amplitude of up-stimulated SOs was significantly higher than down-stimulated SOs from cue presentation to 400 ms after cue offset. These results are in line with findings reported in [9] but the longer sound duration used in this earlier work resulted in the synchronization of up- and down-stimulated SOs into a second SO cycle 400 ms after cue onset (time from which amplitude differences were no longer observed between up- and down-stimulated SOs in [9]).

TF analyses: The results of the TF analyses are presented in Figure S1b-d and indicate that power in the sham conditions was greater than in the stimulated conditions (both up- and down-) between 5 and 10 Hz (up comparison: from -0.9 sec to 0.5 sec, strongest at -0.3 sec - relative to cue; down comparison: from -0.4 sec to 1.3 sec, strongest at -0.1 sec - relative to cue) and between 18 and 22 Hz (up comparison: 0.6 sec post-cue onset; down comparison: 1.2 sec post-cue onset; Figure S1c but see also S10b for TFRs in each condition). Note that in both conditions, these power modulations are in phase with the SO negative peak. As expected, when directly comparing the two stimulation conditions (see Figure S1d), we observed that the power modulations in the 5-10 Hz band and in the sigma band alternated with the phase of the SOs (see details in caption of Figure S1). As up- and down-stimulated SOs in the current study are never in phase (in contrast to [9] where conditions synchronize 400ms after

cue onset into a second SO cycle), the comparison of cue-locked TFRs between conditions brings limited insight into power modulation in our study (beyond phase-related differences shown in Figure S1d). Instead, when phase-related modulations are controlled for (see trough-locked analyses presented in the main text), our results indicate a difference in sigma power between up and down conditions similar as the one reported in [9] on the second (synchronized) SO cycle elicited by the 500-ms acoustic stimulation.

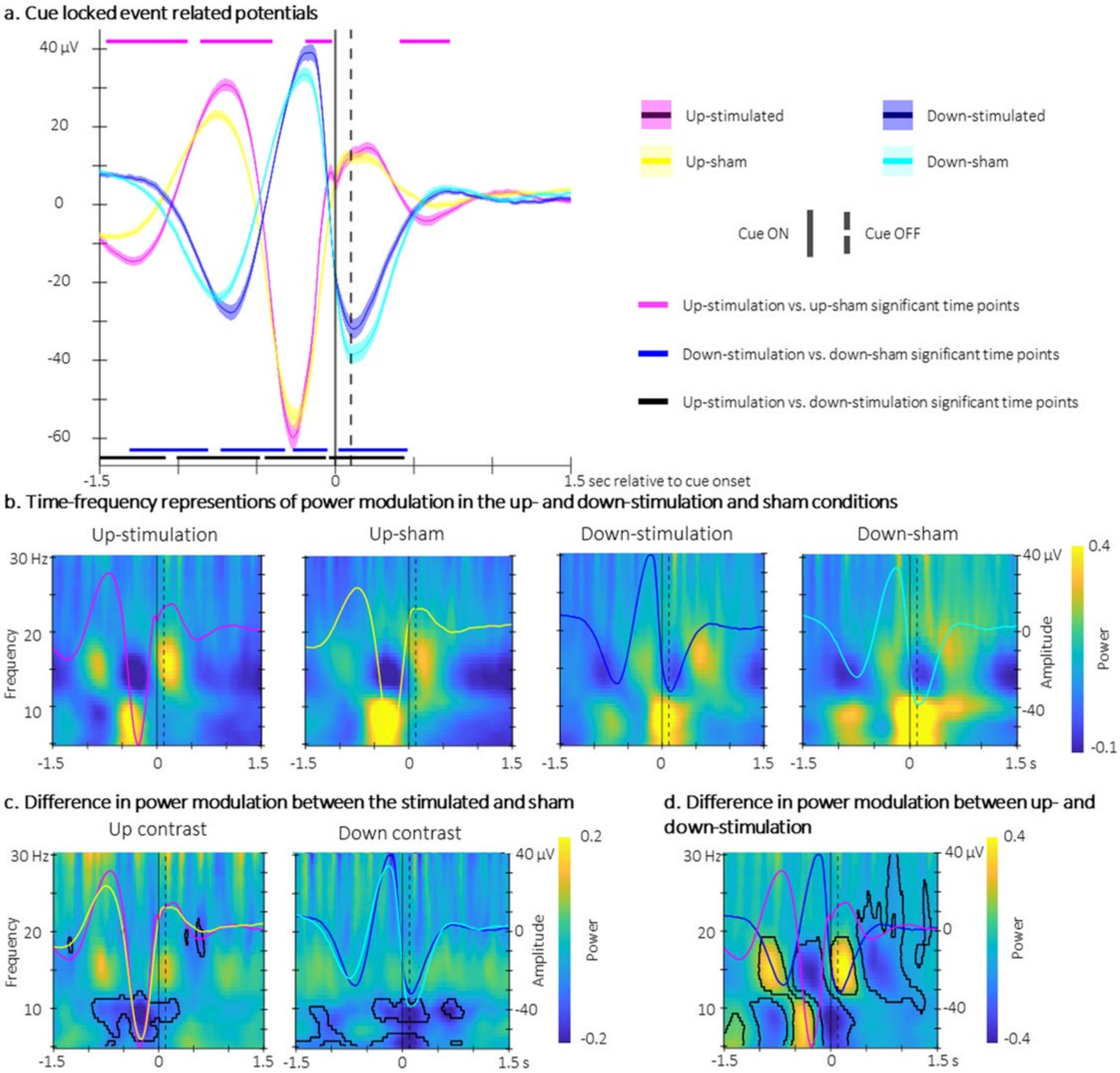

Figure S1: Results of the cue-locked electrophysiological analyses. a. Event-related potentials (n = 30). *ERP grand-average illustrated at Fz (magenta (yellow): true (sham) stimulation in the up condition; blue (cyan): true (sham) stimulation in the down condition). Horizontal lines represent the adjacent time points of the significant spatio-temporal clusters showing a difference in ERP amplitude between conditions. The solid vertical line represents the cue onset and the dashed line represents the cue offset (100ms sound duration). (i) The up-stimulation vs. up-sham contrast (magenta) revealed four clusters whereby SO amplitude was greater in the stimulation as compared to the sham condition. Two clusters showed greater negative ERP amplitude in the stimulation as compared to sham condition (from -1.46 to -0.94 sec and from 0.41 to 0.73 sec relative to cue onset) and two clusters showed greater positive ERP amplitude in the stimulation as compared to the sham condition (from -0.86 to -0.4 sec and from -0.19 to -0.02sec); (ii) The down-stimulation vs. down-sham contrast (blue) revealed three significant clusters pre-cue onset whereby SO amplitude was greater in the stimulation as compared to the sham condition (greater positive amplitude from -0.27 to -0.05 sec and from -1.31 to -0.81 sec; greater negative amplitude from -0.73 to -0.32 sec). An additional cluster was observed at cue onset whereby the amplitude of the negative peak was lower in the stimulation as compared to sham condition (from 0.02 to 0.46 sec); The (iii) up-stimulation vs. down-stimulation contrast (black) revealed four significant clusters in which SO amplitude differed between conditions according to the phase difference between conditions (from -1.5 to -1.08 sec, -1.01 to -0.48 sec, -0.45 to 0.06 sec*

and -0.04 to 0.44 sec). ***b-d. Time-frequency representations.*** *TFRs of the power modulation (as compared to baseline) illustrated at Cz on which the grand-average of the SOs at Fz is super-imposed (same color code as in a).* ***b. Power modulation within each condition. c. Difference in power modulations between stimulation and sham conditions****. TFRs of the difference in power modulation for the three contrasts of interest. The area highlighted in the TFR represents the adjacent time-frequency points of the significant spatio-temporal-frequency cluster showing a difference in power between conditions. The results indicate that power in the sham conditions was greater than in the stimulation condition for both up and down conditions between 5 and 10 Hz (up comparison: from -0.95 to 0.3 sec - strongest at -0.25 sec - relative to cue; down comparison: from -1.5 to -0.68 sec, from -0.42 to 0.4 sec - strongest at 0.1 sec -, and from 0.55 to 0.85 sec relative to cue onset) and between 18 and 22 Hz in the up condition (from -1.28 to -1.24 sec and from 0.54 to 0.65 sec relative to cue onset). These power modulations are in phase with the SO negative peak across conditions.* ***d. Difference in power modulations between up- and down-stimulation****. Results show that power modulations in the 5-10 Hz band and in the 12-18 Hz band alternate with the phase of the SOs. All presented clusters are significant after permutations and after correction for multiple comparisons (cluster p-values < 0.0083). Shaded area around the ERP grand average represents the +/- standard error.*

2. Trough-locked electrophysiological analyses on all channels

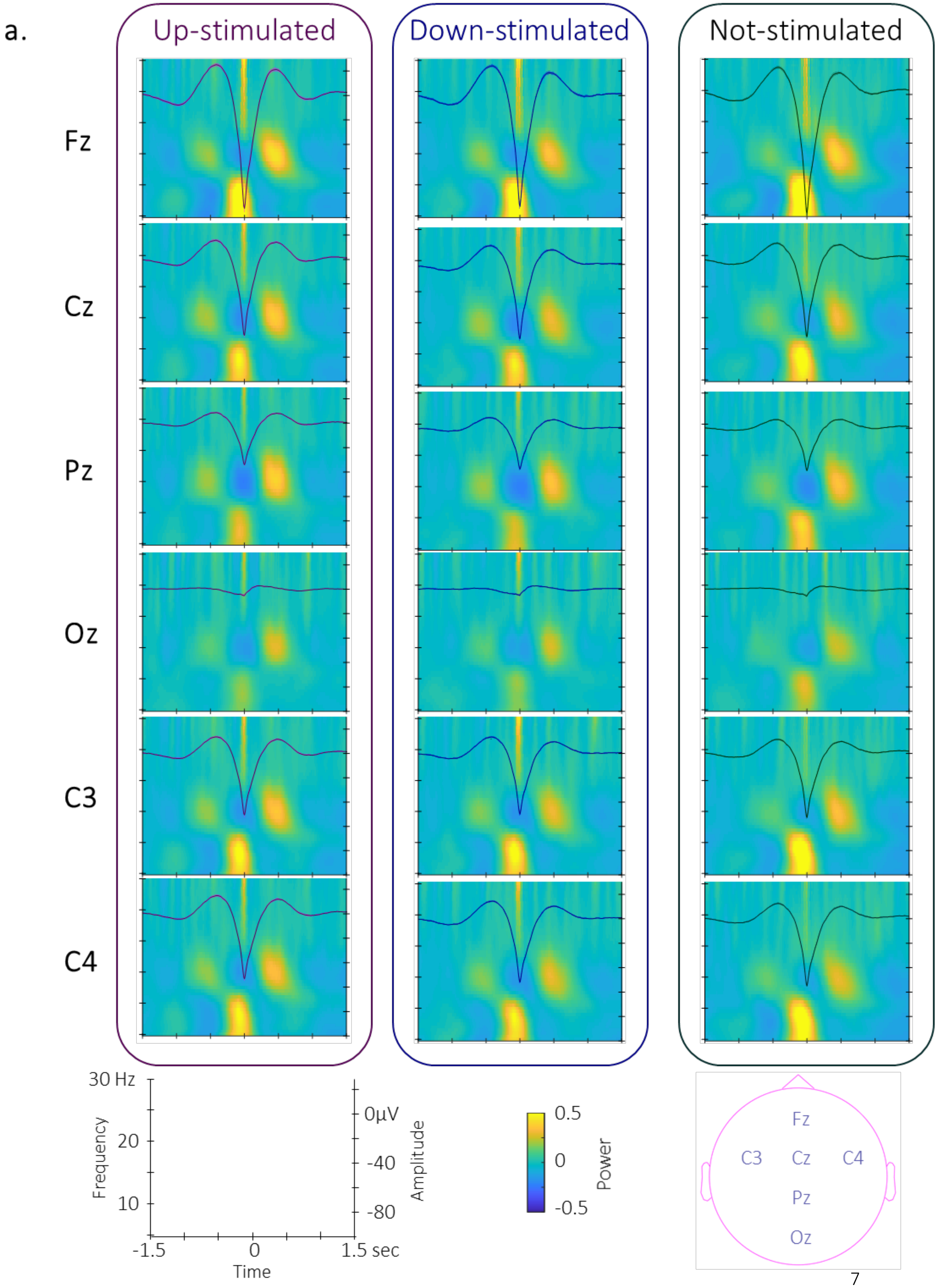

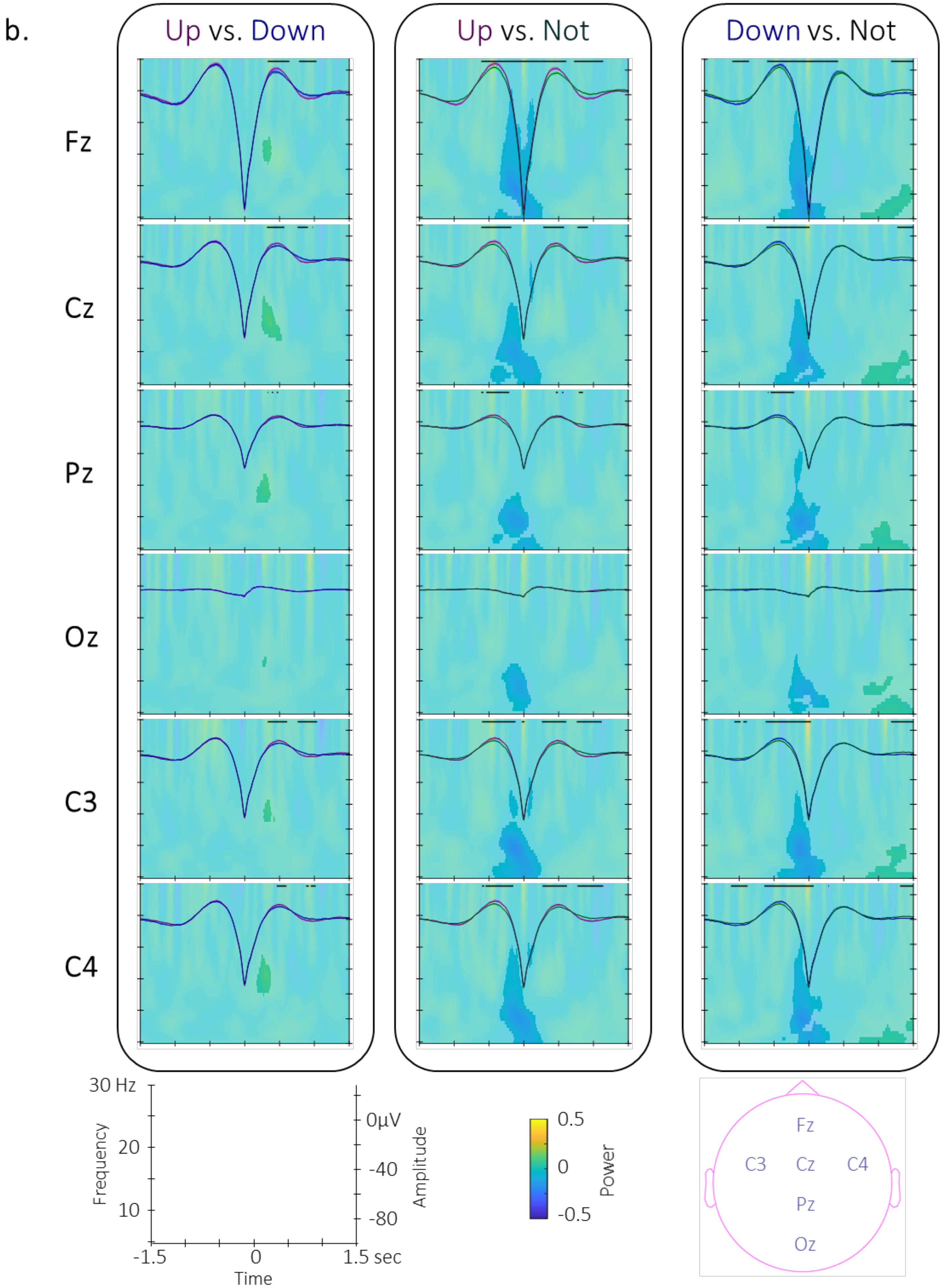
b.
Up vs. Down
Up vs. Not
Down vs. Not
Fz
Cz
Pz
Oz
C3
C4
30 Hz
20
10
Frequency
-1.5
0
1.5 sec
Time
0µV
-40
-80
Amplitude
0.5
0
-0.5
Power
Fz
C3
Cz
C4
Pz
Oz

Figure S2 (previous page): Channel-level EEG data (n = 30). *Statistical comparisons were performed using non-parametric cluster-based permutations.* ***a.*** *Time-frequency representation (TFR) of the group average time-frequency power per condition on which is super-imposed the group average (+/- standard error in shaded regions) of trough-locked SO (**left**: up-stimulated (magenta); **middle**: down-stimulated (blue); **right**: not-stimulated (green);* ***b. Between condition contrasts.*** *Time-frequency representation (TFR) of the difference in power modulation around the trough of the SOs on which the grand-average of the up- vs. down-stimulated SOs is super-imposed (**left**), up- vs. not-stimulated SOs (**middle**), and down- vs. not-stimulated SOs (**right**). As in the main text, black lines represent the adjacent time points of the significant spatio-temporal clusters showing a difference in SO amplitude between the two trough-locked ERPs and highlighted areas in the TFR represents the adjacent time-frequency points of the significant spatio-temporal-frequency cluster showing a difference in power between conditions.*

3. Sleep event detections

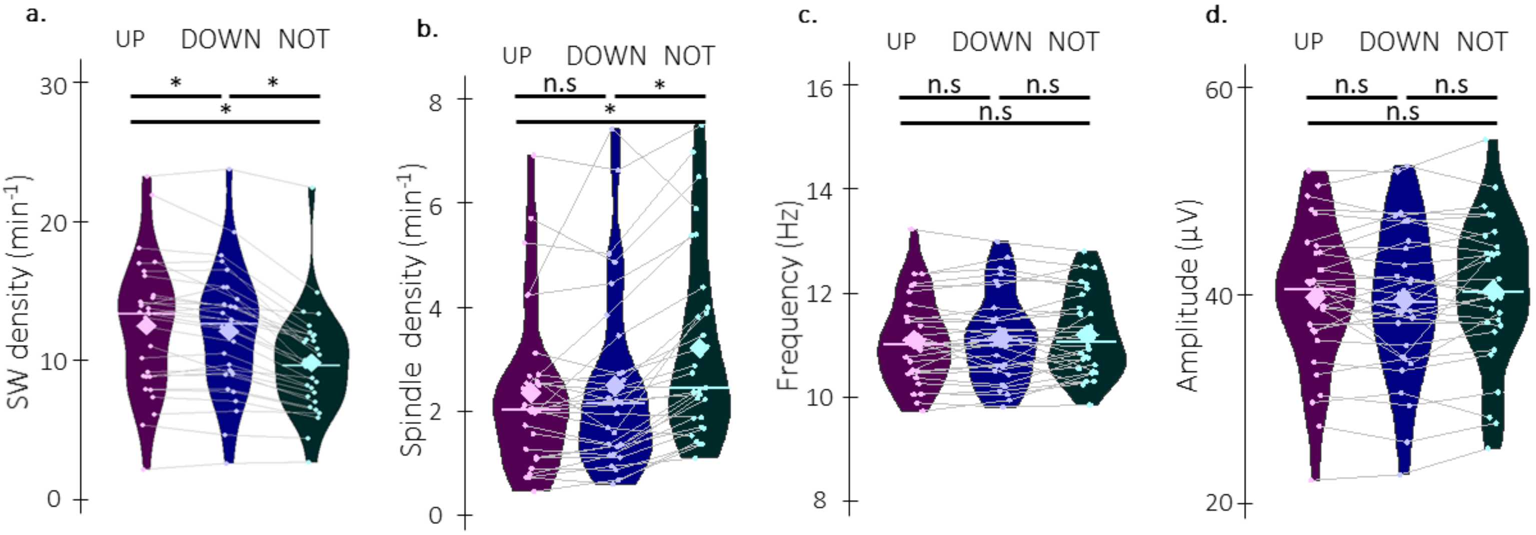

Figure S3: Offline detected sleep events *(n = 30).* ***a. SO density*** *(number of SOs per minute spent in stimulation or rest intervals) extracted from the stimulated and non-stimulated blocks on each of the 6 EEG channels and then averaged across channels was also modulated by the stimulation condition (rmANOVA: main effect of condition: F (2,58) = 52.86, p < 0.001 (< 0.001 sphericity corrected), η^2 = 0.65). SO density was greater during both up- and down-stimulated as compared to non-stimulated blocks (two-sided t-test: up vs. not: t = 7.50, df = 29, p-value < 0.001 (< 0.001 FDR-corrected), Cohen's d = 1.37; down vs. not: t(29) = 8.03, df = 29, p-value < 0.001 (< 0.001 FDR-corrected), Cohen's d = 1.47) and SO density was greater during up- as compared to down-stimulated blocks (up vs. down: t(29) = -2.28, df = 29, p-value = 0.030 (0.030 FDR-corrected), Cohen's d = 0.42).* ***b. Spindle density*** *was modulated by the intervention (rmANOVA: condition effect: F (2,58) = 22.34, p < 0.001 (< 0.001 sphericity corrected), η^2 = 0.44), such that density was lower during both up- and down-stimulated as compared to non-stimulated blocks, irrespective of the phase of the stimulation (two-sided t-tests: up vs. down: t = 0.97, df = 29, p-value = 0.34 (0.34 FDR-corrected), Cohen's d = 0.18; up vs. not: t = -6.16, df = 29, p-value < 0.001 (< 0.001 FDR-corrected), Cohen's d = 1.12; down vs. not: t = -5.41, df = 29, p-value < 0.001 (< 0.001 FDR-corrected), Cohen's d = 0.99).* ***c. Spindle frequency*** *did not statistically differ among stimulation conditions (rmANOVA: F (2,58) = 1.50, p = 0.23 (0.23 sphericity corrected), η^2 = 0.050).* ***d. Spindle amplitude*** *also did not statistically differ among stimulation conditions (rmANOVA: F (2,58) = 2.56, p = 0.086 (0.093 sphericity corrected), η^2 = 0.081). Violin plots: median (horizontal bar), mean (diamond), the shape of the violin plots depicts the distribution of the data. Colored points represent individual data, jittered in arbitrary distances on the x-axis within the respective violin plot to increase perceptibility. n.s. = non-significant; *: p-value < 0.05.*

4. Main effect of practice (across sequences) and its linear modulation by performance

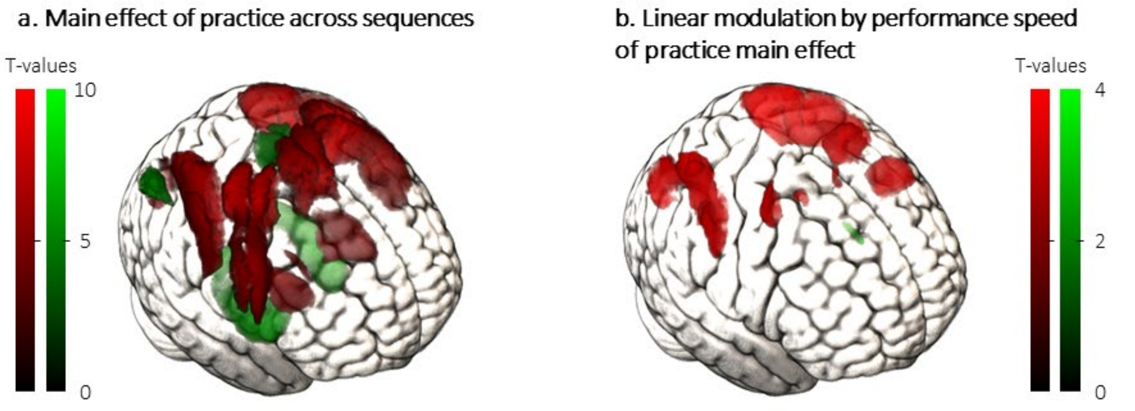

*Figure S4: **Brain activity elicited by the practice of the motor sequence learning task** (n = 28). **a. Main effect of practice across sequences.** Task practice – as compared to rest - resulted in greater (red) activity in striatal and motor cortical regions and in less (green) activity the bilateral hippocampus and the supplementary motor area. **b. Linear modulation of brain activity as function of changes performance during task practice.** Brain activity decreased (red) with performance improvement in a network including motor and parietal regions while it increased (green) in the caudate. Activation maps are displayed on a T1-weighted template image as part of the MRIcroGL software (https://www.mccauslandcenter.sc.edu/mricrogl) with a threshold of p < 0.005 uncorrected.*

Table S1: Functional imaging results of the main effect of motor sequence learning. The significance threshold was set at $p_{uncorrected} < .005$.

Brain Area	Cluster size	x	y	z	Z
**1. Practice vs. Rest**					
[+T]					
L M1	1891	-38	-36	42	6.93
L M1	2224	-40	-10	62	6.90
L SMA	3239	-10	-6	60	6.55
R aSPL	1965	28	-54	60	6.53
R Putamen	775	24	-2	12	4.92
L Putamen	863	-22	-4	10	4.79
[-T]					
R Hippocampus	1021	28	-14	-20	6.29
L Hippocampus	1146	-28	-20	-16	6.17

R aSPL	200	46	-72	42	5.68
L aSPL	216	-44	-64	44	5.28
L SMA	324	0	-34	54	4.33
L SMA	44	-12	-32	60	3.96
**2. Practice vs. Rest modulated by performance speed**					
[+T]					
R aSPL	1425	34	-72	26	4.58
L aSPL	1793	-24	-62	50	4.45
L PMC	263	-28	-6	60	3.71
L PMC	368	-50	8	26	3.68
R M1	178	30	-6	52	3.45
L SMA	22	-10	4	50	3.19
[-T]					
L Caudate	22	-12	6	22	2.98

5. Activation-based results reported in the main text

Table S2: Functional imaging results for the activation-based analyses. The significance threshold was set at $p_{uncorrected} < .005$.

Brain Area	Cluster size	x	y	z	Z
**1. Changes in brain activity between sessions within stimulation conditions**					
**1.1. Up**					
[Post – Pre]					
R Putamen	1983	24	18	-10	5.33
R Caudate		10	-2	14	4.11
L SMA	1694	-6	-12	56	5.00
L M1	860	-38	-20	52	4.35
R M1	1153	22	-26	52	4.28
L Putamen	2008	-24	-8	-2	4.26
L Caudate		-6	14	2	3.80
R M1	41	30	-38	60	3.58
L aSPL	100	-40	-62	40	3.17
L M1	37	-60	2	16	3.03
L aSPL	37	-56	-26	46	3.00
L aSPL	10	-56	-32	32	2.97
R aSPL	16	58	-18	28	2.94
L M1	8	-22	-14	72	2.86
[Pre – Post] No suprathreshold clusters					
**1.2. Down**					
[Post – Pre]					
L SMA	2173	-8	-12	56	4.61
L M1	1171	-38	-24	54	4.52
R PMC	477	56	14	26	4.23
R Putamen	594	26	-8	-4	4.03
R aSPL	111	66	-16	28	3.95
R M1	88	30	-38	60	3.83
L Putamen	431	-36	0	2	3.60
L aSPL	251	-60	-30	34	3.52
R Caudate	1019	20	22	-8	3.29
R M1	114	42	-4	44	3.18

L M1	19	-30	-38	52	3.10
L SMA	6	-16	-14	60	2.94
R Caudate	34	10	-2	14	2.89
[Pre – Post]					
R Hippocampus	7	32	-38	-6	2.80
1.3. Not					
[Post – Pre]					
L SMA	1366	-2	-4	46	4.65
L Putamen	794	-30	-14	10	4.29
R PMC	244	56	8	8	4.13
R Putamen	557	28	-12	-6	3.88
L M1	560	-38	-26	56	3.71
R M1	400	34	-22	54	3.53
R Caudate	138	10	6	12	3.35
R aSPL	29	58	-18	28	3.23
R PMC	92	50	0	52	3.11
L aSPL	61	-58	-32	34	3.10
L aSPL	58	-42	-62	44	3.06
[Pre – Post]					
R Hippocampus	54	34	-38	-6	3.67
L Hippocampus	12	-34	-40	-8	2.97

Brain Area	Cluster size	x	y	z	Z
2. Differences between conditions in inter-session changes in brain activity					
2.1. Up vs. Down					
[Post – Pre]					
R Caudate	199	20	18	12	3.41
R Putamen		22	4	12	3.00
[Pre – Post] No suprathreshold clusters					
2.2. Up vs. Not					
[Post – Pre]					
R Caudate	60	18	28	4	3.12
L Caudate	11	-18	8	22	3.08

R Caudate	7	4	16	6	3.02
R Hippocampus	14	36	-36	-4	2.98
R Caudate	17	18	4	24	2.96
[Pre – Post] No suprathreshold clusters					
2.3. Down vs. Not					
[Post – Pre]					
R M1	54	22	-28	54	3.42
L aSPL	74	-36	-46	34	3.31
R Caudate	10	16	-2	26	3.22
R aSPL	41	36	-42	34	3.19
R SMA	39	20	-12	56	3.08
[Pre – Post] No suprathreshold clusters					

Brain Area	Cluster size	x	y	z	Z
3. Regressions between overnight increase in brain activity and tmr index					
3.1. Up x TMR up					
[Negative correlation] No suprathreshold clusters					
[Positive correlation]					
L Putamen	1601	-22	20	-4	4.08
L Caudate		-18	24	10	4.06
L Putamen		-28	8	8	4.05
R PMC	226	54	12	20	4.03
R Putamen	1457	30	10	6	4.03
R Caudate		18	0	18	3.85
R Caudate		20	18	12	3.67
R Hippocampus	31	36	-38	-8	3.64
R Hippocampus		40	-28	-12	2.85
L Caudate	49	-4	6	0	3.59
L M1	24	-24	-30	52	3.40
L M1	34	-40	-2	30	3.36
R Hippocampus	26	18	-34	8	3.33
R SMA	468	8	-4	50	3.30
R M1	135	40	-10	52	3.23

L SMA	29	-10	-8	70	3.19
R aSPL	80	48	-36	40	3.08
R M1	85	30	-38	58	3.05
L M1	61	-28	-12	68	2.97
L SMA	10	-20	-12	54	2.94
L Hippocampus	35	-30	-44	0	2.94
R M1	35	28	-20	66	2.89
R M1	14	52	-20	32	2.87
R Hippocampus	5	40	-20	-14	2.85
R Putamen	16	34	-8	-6	2.82
R M1	10	32	-38	38	2.77
**3.2. Down x TMR down**					
[Negative correlation] No suprathreshold clusters					
[Positive correlation]					
R Caudate	65	16	-2	26	3.07
L Hippocampus	24	-16	-36	6	2.87
R Hippocampus	43	20	-34	4	2.84
R SMA	12	4	2	58	2.71

Brain Area	Cluster size	x	y	z	Z
**4. Regressions between overnight increase in brain activity and eeg**					
**4.1. Up x sigma power up**					
[Negative correlation]					
R PMC	11	56	8	10	2.94
L M1	17	-50	-8	38	2.87
[Positive correlation] No suprathreshold clusters					
**4.2. Down x sigma power down**					
[Negative correlation] No suprathreshold clusters					
[Positive correlation]					
L Caudate	15	-10	18	-6	2.72
**4.3. Up x peak amplitude up**					

[Negative correlation] No suprathreshold clusters					
[Positive correlation]					
R Pallidum	40	18	-4	-4	3.49
R aSPL	5	56	-42	54	2.85
L M1	12	-46	-16	48	2.72

4.4. Down x peak amplitude down					
[Negative correlation] No suprathreshold clusters					
[Positive correlation]					
L M1	89	-24	-20	68	3.34
L M1	94	-44	-16	52	3.33
L aSPL	33	-62	-20	40	3.09
L M1	21	-46	-10	26	3.02
L M1	32	-20	-16	70	2.97
R M1	11	48	-8	26	2.86
R M1	9	44	-14	52	2.78

6. Connectivity results reported in the main text

Table S3: Functional imaging results for the connectivity analyses. The significance threshold was set at $p_{uncorrected} < .005$.

Brain Area	Cluster size	x	y	z	Z
**1. Overnight changes in connectivity within conditions**					
**1.1. Up**					
**1.1.1. Right caudate**					
[Post – Pre] No suprathreshold clusters					
[Pre – Post] No suprathreshold clusters					
**1.1.2. Right putamen**					
[Post – Pre] No suprathreshold clusters					
[Pre – Post] No suprathreshold clusters					
R Putamen	14	30	12	-2	2.81
**1.1.3. Right hippocampus**					
[Post – Pre] No suprathreshold clusters					
[Pre – Post]					
R PMC	25	40	4	26	3.15
R PMC	100	42	-4	56	3.08
R aSPL	14	36	-66	28	3.00
L M1	21	-38	-4	56	2.74
R aSPL	25	32	-52	52	2.72
**1.2. Down**					
**1.2.1. Right caudate**					
[Post – Pre] No suprathreshold clusters					
[Pre – Post] No suprathreshold clusters					
**1.2.2. Right putamen**					
[Post – Pre]					
L M1	28	-46	-8	26	3.03

R M1	17	28	-8	44	2.92
L M1	5	-52	2	48	2.71
L M1	11	-32	-18	48	2.69
L SMA	13	-22	-12	58	2.67
[Pre – Post] No suprathreshold clusters					

1.2.3. Right hippocampus

[Post – Pre] No suprathreshold clusters

[Pre – Post]

L M1	7	-60	-4	22	2.73

1.3. Not

1.3.1. Right caudate

[Post – Pre]

L Hippocampus	7	-32	-20	-14	2.70
[Pre – Post] No suprathreshold clusters					

1.3.2. Right putamen

[Post – Pre]

R Ventral striatum	18	14	18	-10	3.02
[Pre – Post] No suprathreshold clusters					

1.3.3. Right hippocampus

[Post – Pre] No suprathreshold clusters

[Pre – Post] No suprathreshold clusters

Brain Area	Cluster size	x	y	z	Z

2. Overnight changes in connectivity between conditions

2.1. Up vs. Down

2.1.1. Right caudate

[Post – Pre] No suprathreshold clusters

[Pre – Post]

R aSPL	16	54	-36	56	2.80

2.1.2. Right putamen

[Post – Pre] No suprathreshold clusters

[Pre – Post]

R aSPL	220	28	-46	42	3.60
L M1	28	-22	-14	52	3.17
L M1	96	-32	-20	50	3.15
R Putamen	45	26	12	-4	2.93
L SMA	13	-10	-10	54	2.87

2.1.3. Right hippocampus

[Post – Pre] No suprathreshold clusters

[Pre – Post] No suprathreshold clusters

2.2. Up vs. not

2.2.1. Right caudate

[Post – Pre] No suprathreshold clusters

[Pre – Post]

L aSPL	89	-50	-50	48	2.97
R aSPL	32	50	-36	56	2.77

2.2.2. Right putamen

[Post – Pre] No suprathreshold clusters

[Pre – Post] No suprathreshold clusters

2.2.3. Right hippocampus

[Post – Pre] No suprathreshold clusters

[Pre – Post]

L M1	141	-26	-22	54	3.46
R SMA	268	12	-12	48	3.36
R M1	181	60	-24	48	3.19
L M1	76	-26	-14	54	3.05
R SMA	74	22	-8	52	3.02
L aSPL	262	-40	-42	48	2.96

R M1	35	28	-38	54	2.91
R M1	36	48	-10	52	2.80
R M1	21	24	-28	54	2.79
R SMA	23	14	-4	68	2.69
L aSPL	7	-26	-52	54	2.69

2.3. Down vs. not

2.3.1. Right caudate

[Post – Pre] No suprathreshold clusters

[Pre – Post]

L Pallidum	9	-22	-14	-4	2.81

2.3.2. Right putamen

[Post – Pre] No suprathreshold clusters

[Pre – Post] No suprathreshold clusters

2.3.3. Right hippocampus

[Post – Pre] No suprathreshold clusters

[Pre – Post]

L SMA	174	0	-34	54	3.59
R M1	30	22	-14	56	3.21
L aSPL	369	-30	-46	44	3.12
L M1	100	-54	-10	44	3.10
L M1		-48	-10	26	3.05
R M1	60	48	-10	34	3.00
R M1	55	54	-22	46	2.85
L Caudate	5	-18	-14	20	2.74
R SMA	7	12	-12	46	2.72
R M1	9	62	-2	36	2.69

Brain Area	Cluster size	x	y	z	Z
**3. Regression between overnight increases in connectivity within condition and TMR index**					
**3.1. Up x TMR up**					
**3.1.1. Right caudate**					
[Negative correlation]					
R aSPL	99	44	-46	50	2.84
[Positive correlation] No suprathreshold clusters					
**3.1.2. Right Putamen**					
[Negative correlation]					
R aSPL	38	44	-42	44	2.78
R aSPL	8	38	-60	42	2.65
[Positive correlation]					
R Pallidum	9	22	-8	-6	2.95
L Caudate	13	-16	6	22	2.86
**3.1.3. Right hippocampus**					
[Negative correlation]					
R aSPL	77	40	-70	34	3.71
R Putamen	61	32	-8	6	3.10
R SMA	16	8	-20	62	2.82
[Positive correlation]					
R Hippocampus	8	28	-36	-4	2.74
**3.2. Down x TMR down**					
**3.2.1. Right caudate**					
[Negative correlation] No suprathreshold clusters					
[Positive correlation]					
L Hippocampus	46	-16	-40	6	3.30
R Caudate	29	16	-2	26	3.24
R Ventral striatum	10	4	6	0	2.85

R Hippocampus	8	18	-38	6	2.72

3.2.2. Right Putamen

[Negative correlation]

R Putamen	21	28	-2	14	3.34
R M1	11	26	-8	44	3.09
R Caudate	7	12	-14	20	2.87

[Positive negative correlation] No suprathreshold clusters

3.2.3. Right hippocampus

[Negative correlation]

[Positive correlation]

Brain Area	Cluster size	x	y	z	Z

4. Regression between overnight increases in connectivity within condition and eeg

4.1. Up x Sigma power up

4.1.1. Right caudate

[Negative correlation]

R aSPL	60	44	-40	38	3.41
R aSPL	10	36	-68	42	2.70

[Positive correlation]

L SMA	135	-10	-22	58	3.41
R Caudate	17	14	24	10	3.36
R Hippocampus	27	38	-14	-22	3.33
R Ventral striatum	15	6	8	-6	3.30
R Putamen	20	24	8	-12	2.80

4.1.2. Right putamen

[Negative correlation] No suprathreshold clusters

[Positive correlation]

L Caudate	16	-18	24	10	3.13

4.1.3. Right hippocampus

[Negative correlation]					
R M1	22	26	-4	48	3.15
[Positive correlation] No suprathreshold clusters					

4.2. Down x Sigma power down

4.2.1. Right caudate

[Negative correlation]					
L Hippocampus	102	-24	-14	-8	3.68
R Ventral striatum	35	12	6	-8	3.65
R Hippocampus	47	20	-18	-24	3.55
L Ventral striatum	41	-8	8	-12	3.49
L aSPL	52	-38	-70	44	3.41
R aSPL	54	40	-72	48	3.26
L Putamen	27	-34	4	-8	3.20
[Positive correlation] No suprathreshold clusters					

4.2.2. Right putamen

[Negative correlation]					
L Hippocampus	145	-18	-10	-8	3.25
[Positive correlation] No suprathreshold clusters					

4.2.3. Right hippocampus

[Negative correlation] No suprathreshold clusters

[Positive correlation] No suprathreshold clusters

4.3. Up x peak amplitude up

4.3.1. Right caudate

[Negative correlation] No suprathreshold clusters

[Positive correlation] No suprathreshold clusters

4.3.2. Right putamen

[Negative correlation] No suprathreshold clusters

[Positive correlation] No suprathreshold clusters

4.3.3. Right hippocampus

[Negative correlation] No suprathreshold clusters

[Positive correlation] No suprathreshold clusters

4.4. Down x peak amplitude down

4.4.1. Right Caudate

[Negative correlation]

R Hippocampus	1276	14	-30	-10	4.62
L Hippocampus	950	-16	-8	-16	4.26
R PMC	2573	50	-2	54	4.25
R SMA		6	2	64	4.24
L M1	1230	-62	-20	28	3.90
L aSPL		-44	-44	38	3.84
L M1	401	-62	4	16	3.67
L M1		-34	-4	42	3.36
L Caudate	61	-6	2	0	3.54
R M1	31	62	-2	14	3.35
L M1	192	-24	-28	58	3.32
R aSPL	21	66	-16	28	3.31
R Caudate	35	12	-8	18	3.26
R M1	464	26	-46	46	3.18
R PMC	12	56	8	8	3.14
L M1	73	-44	-12	44	2.99
L Caudate	17	-14	20	6	2.89
R Ventral striatum	6	2	8	-2	2.86
L Putamen	9	-26	-18	10	2.82
R Caudate	6	8	14	8	2.72

[Positive correlation] No suprathreshold clusters

4.4.2. Right putamen

[Negative correlation] No suprathreshold clusters

L aSPL	1738	-36	-42	36	4.56
R aSPL	467	34	-42	48	3.93
R Hippocampus	170	32	-22	-12	3.74
R aSPL	29	30	-66	38	3.56

R SMA	140	12	-2	66	3.55
R M1	143	58	12	36	3.54
R PMC	121	28	-12	60	3.16
L M1	125	-48	4	32	3.15
R M1	31	40	0	46	3.06
R Hippocampus	22	26	-38	-6	3.01
R SMA	27	8	4	48	2.98
R aSPL	18	34	-66	58	2.94
L Pallidum	28	-16	-2	-2	2.92
R Hippocampus	5	30	-4	-28	2.86
L Putamen	7	-26	-14	12	2.79
L M1	18	-28	-14	62	2.75
L Hippocampus	7	-26	-36	-10	2.70
[Positive correlation] No suprathreshold clusters					

4.4.3. Right hippocampus

[Negative correlation]					
L SMA	190	-6	-28	56	3.42
L PMC	111	-50	8	16	3.17
L M1	53	-32	-18	42	3.17
L aSPL	67	-46	-56	46	2.80
R Caudate	42	8	16	12	3.05
L Caudate	100	-8	6	14	3.03
L Caudate		-12	14	6	2.94
L M1	75	-24	-28	58	2.98
R SMA	32	18	-26	58	2.95
R M1	11	56	-2	18	2.71
[Positive correlation] No suprathreshold clusters					

7. Brain-behavior regression analyses using between-condition activation maps

In addition to the regression analyses presented in the main text between the individuals' brain maps showing between session changes in activity/connectivity ***within*** each condition and the individuals' TMR index, we performed similar regression analyses but using maps showing between session changes in activity/connectivity ***between*** conditions. As detailed below, these supplementary analyses yielded similar results as those presented in the main text with the *within-condition* contrasts.

For the activation-based contrasts, results show that the correlation reported in the original manuscript (see Figure 4c and 5c in main manuscript) for both up and down conditions between the overnight changes in striato-motor and hippocampal activity and the TMR index was replicated with the between-condition contrasts (Figure S5a-d). Specifically, the present analyses show that the between-session increase in striato-motor activity that was greater in the reactivated (both up and down) as compared to the not-reactivated condition was related to a greater TMR index (Figure S5a,c-d). Additionally, the initial correlation between the overnight changes in hippocampal activity and the TMR index observed for both the up- and down-reactivated sequences (Figure 5c in the main manuscript) was replicated with the between-condition contrast 'up vs. not'. Here, the overnight decrease in activity – that was greater for the not-reactivated as compared to the up-reactivated condition - was related to a lower TMR index (Figure S5b).

For the connectivity-based contrasts, results show that the correlation reported in the main manuscript (see Figure 6c and 7d) for both up and down conditions between the overnight changes in striato-hippocampal connectivity and the TMR index was replicated with the between-condition contrasts (Figure S5e-f). Specifically, the new analyses show that the between-session decrease in striato-hippocampal connectivity – that was greater for the up- as compared to the not-reactivated sequence - was related to a greater TMR index (Figure S5e). Additionally, the initial correlation between the overnight increase in striato-hippocampal connectivity and the TMR index observed for the down-reactivated sequence (Figure 7d in the main manuscript) was also replicated with the between-condition contrast. Here, a greater difference in connectivity increase between the down- and not-reactivated conditions was related to a greater TMR index (Figure S5f).

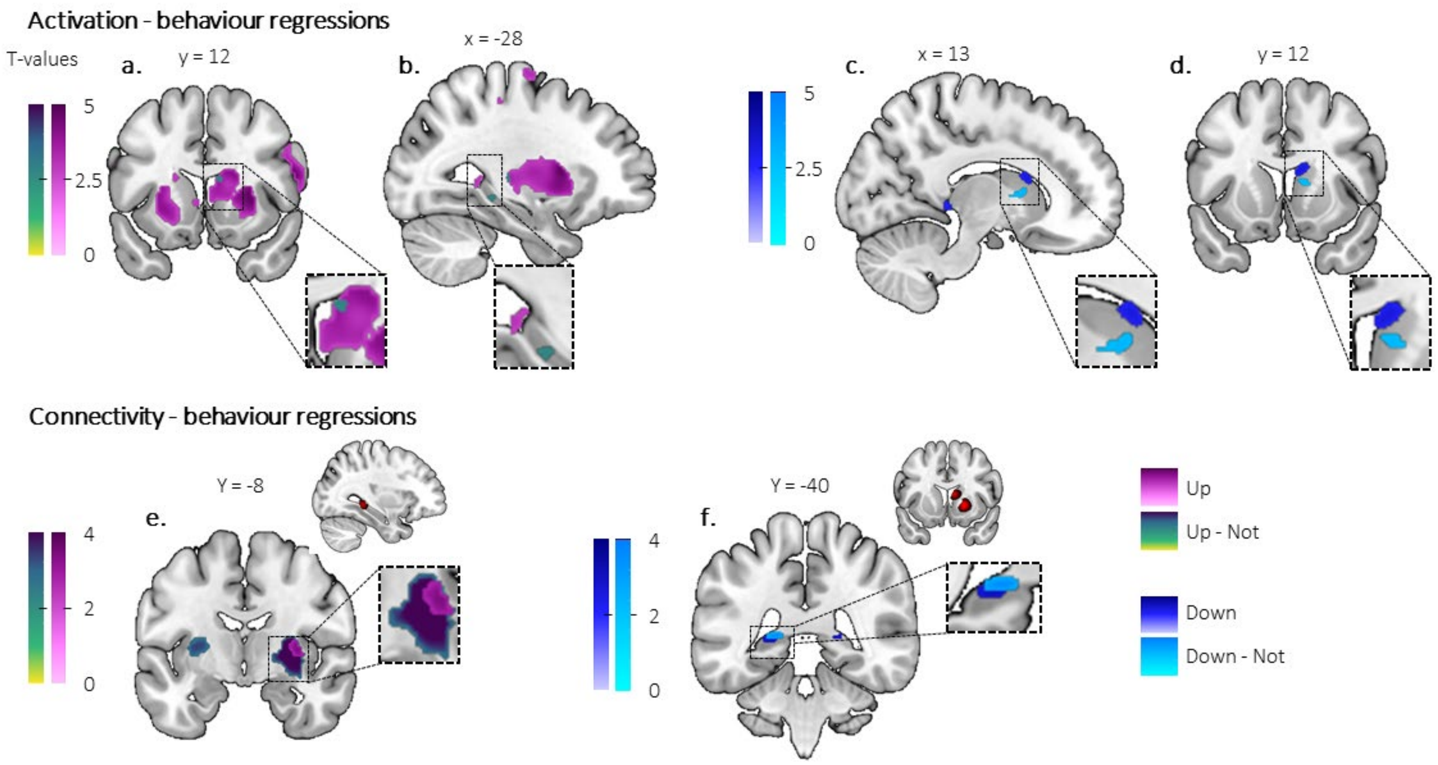

Figure S5: Overlay of the within-condition and between-condition contrasts regression analyses (n = 28). *In each plot, the significant clusters of the within-condition regression analyses (magenta or blue for up- or down-reactivated conditions, respectively) are overlaid with the results of the between-condition regression analyses in which the TMR index is regressed against the difference in BOLD response between the reactivated and not-reactivated conditions between sessions. Activations maps are displayed on a T1-weighted template image with a threshold of $p < 0.005$ uncorrected.* ***a****. Regressions between the overnight increase in striatal activity for the up-reactivated (as compared to the not-reactivated) sequence and the TMR_{Up} index.* ***b.*** *Regressions between the overnight decrease in hippocampal activity for the up-reactivated (as compared to the not-reactivated) sequence and the TMR_{Up} index.* ***c-d.*** *Regressions between the overnight increase in striatal activity for the down-reactivated (as compared to the not-reactivated) sequence and the TMR_{Down} index.* ***e****. Regression between the overnight decrease in connectivity for the up-reactivated sequence (as compared to the not-reactivated) and the TMR_{Up} index.* ***f****. Regression between the overnight decrease in connectivity for the down-reactivated sequence (as compared to the not-reactivated) and the TMR_{Down} index. Activation maps are displayed on a T1-weighted template image as part of the MRIcroGL software (https://www.mccauslandcenter.sc.edu/mricrogl) with a threshold of $p < 0.005$ uncorrected.*

8. Supplementary regressions analyses between connectivity and performance/sleep measures

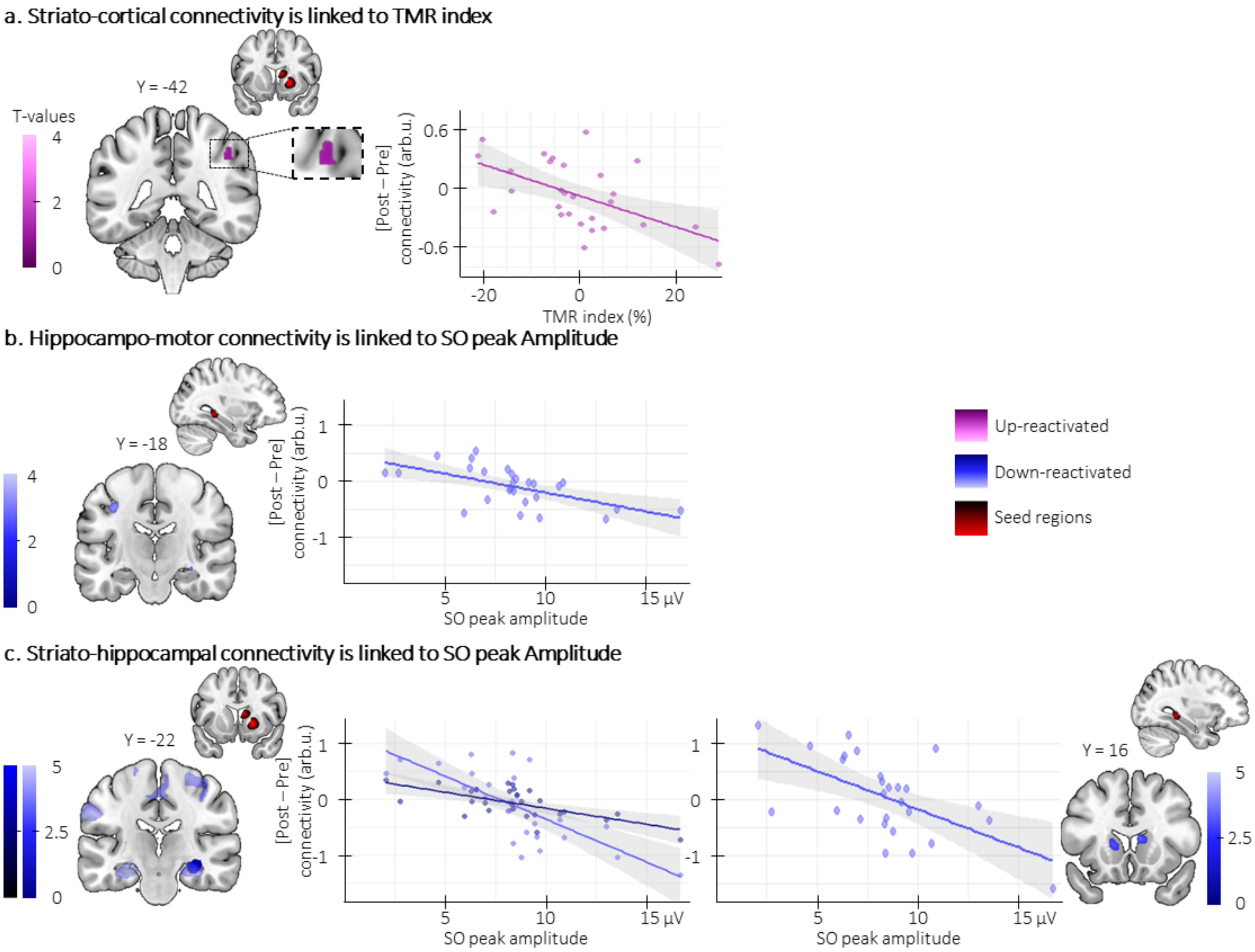

Figure S6: Regressions between connectivity, behavior and EEG metrics (n = 27 unless specified otherwise). Statistical parametric maps were generated using a general linear model in SPM12 (see methods). ***a Striato-cortical connectivity and TMR index*** *(n = 28). The overnight decrease in striato-motor connectivity for the up-reactivated sequence was linked to the TMR index (putamen-right aSPL: x = 44, y = -42 z = 44, Z = 2.78, p_{SVC} = 0.053) such that the greater the decrease in connectivity (y-axis), the greater the performance improvement on the up-reactivated as compared to the not-reactivated sequence (x-axis).* ***b. Hippocampo-motor connectivity and SO peak amplitude.*** *The overnight decrease in hippocampo-motor connectivity for the down-stimulated condition was linked to the SO peak amplitude (hippocampus-left M1: x = -32, y = -18, z = 42, Z = 3.17, p_{SVC} = 0.02) such that the greater the decrease in connectivity (y-axis), the greater the SO peak amplitude during the night(x-axis).* ***c. Striato-hippocampal connectivity and SO peak amplitude.*** *The overnight increase in striato-hippocampal connectivity in the down-stimulated condition was linked to the SO peak amplitude (**left panel**, Caudate-right hippocampus (pale blue): x = 14, y = -32, z = -8, Z = 4.62, p_{SVC} < 0.001; putamen-right hippocampus (dark blue): x = 32, y = -22, z = -12, Z = 3.74, p_{SVC} = 0.004; **right panel**, hippocampus-right caudate: x = 8, y = 16, z =12, Z = 3.05, p_{SVC} = 0.027) such that the greater the increase in connectivity (y-axis), the lower the SO peak amplitude during the night (x-axis). Activations maps are displayed on a T1-weighted template image with a threshold of p < 0.005 uncorrected. arb.u.: arbitrary units. Violin plots: median (horizontal bar), mean (diamond), the shape of the violin plots depicts the kernel density estimate of the data. Shaded area around the linear regressions represents the 95% confidence interval. Activation maps are displayed on a T1-weighted template image as part of the MRIcroGL software (https://www.mccauslandcenter.sc.edu/mricrogl) with a threshold of p < 0.005 uncorrected.*

List of the deviations from the pre-registration

Table S4. List of the deviations from the pre-registered analyses followed by their justification. These deviations are marked with a [#] in the methods of the main manuscript.

	Pre-registered	Final report
1	Task-based functional volumes of each participant will be realigned to the first image of each session and then realigned to the mean functional image computed across MRI runs of the respective participant using rigid body transformations **and corrected for inhomogeneities in the magnetic field (i.e., unwarped) using the field map images**.	Additionally, to correct for distortions due to magnetic field inhomogeneities, field maps (TR = 1500 ms; TE = 3.5 ms; flip angle = 90°; 42 transverse slices; slice thickness = 3.75 mm; interslice gap = 0 mm; voxel size = 3.75 × 3.75 × 3.75 mm3; field of view = 240 × 240 × 157.5 mm3; matrix = 64 × 64) were collected […]. **However, these sequences were not included in the final analysis pipeline.**
	**Justification**: The introduction of the field maps in the processing pipeline induced unforeseen image distortions in some participants. We therefore elected to not use the field maps in the final analysis pipeline in order to keep the preprocessing consistent across individuals.	
2	Individual trials (i.e., reaction times to visual stimuli during the MSL tasks) will be excluded from further analysis if the trial deviates more than three standard deviations from the block's average reaction time.	Correct trials were excluded from the analyses if they were outlier trials based on **John Tukey's method of leveraging the Interquartile**.
	**Justification**: The John Tukey's method of leveraging the Interquartile was used for outlier exclusion as the median was the statistical average used in this study.	
3	The power in the sigma band will be compared between the reactivated-up and the control SWs and between the reactivated-down and the control SWs separately using cluster-based permutation tests (Maris & Oostenveld, 2007) using an alpha threshold at 0.025 (Bonferonni corrected for two comparisons).	To identify significant evoked power modulation, TFR locked to the SO trough were compared 2-by-2 using a CBP tests between 5 to 30 Hz and from -1.5 to 1.5 sec relative to cue onset to exclude border effects and **Bonferonni corrected for three multiple comparisons**.
	**Justification**: For completeness, we decided to also compare the two stimulated conditions. Therefore, the number of tests was increased to 3 and the Bonferroni correction needed to be adjusted.	
4	*SW-spindle coupling*: Finally, we aim to determine whether the amplitude of the oscillatory signal is coupled to the phase of the delta band using the tensorPac[1] open-source Python toolbox. The event-related PAC will be computed in relation to the trough of the SWs.	Analysis not performed.
	**Justification**: As per the inclusion of exploratory analyses that focused on trough-locked oscillatory activity modulation, the phase-amplitude coupling analysis became redundant.	
5	We will extract the frequency and the amplitude of spindles as well as the **amplitude** and the density of both spindles and SO.	We extracted the frequency and the amplitude of spindles as well as the density of both spindles and SO.
	**Justification**: We substitute the pre-registered SO amplitude analysis performed on the discrete sleep events with a more comprehensive (i.e., across all time points in the analysis window) and conservative trough-locked potential analysis using the same approach as the pre-registered oscillatory activity analysis, i.e., using Cluster-based permutation approach. Nevertheless, the corresponding hypotheses remained unchanged. Namely, (i) up-stimulated SO amplitude will be boosted as compared to down- and not-stimulated SO whereas (ii) down-stimulated SO amplitude will be impaired as compared to up- and not-stimulated SOs.	
6	Linear contrasts will be generated at the individual level to test for (1) the main effect of practice (across sequences) and its linear modulation by performance, (2) the main effect of practice for each sequence (reactivated-up, reactivated-down, and non-reactivated) and their linear modulation by performance and (3)	Pre-night test linear contrasts are not reported in the present study.

<table>
<tr><td></td><td>difference in brain responses between sequences practiced (reactivated-up vs. reactivated-down vs. non-reactivated) and their linear modulation by performance. These contrasts will be written within each task run (pre-night training, pre-night test, post-night training) as well as between task runs.</td><td></td></tr>
<tr><td></td><td colspan="2">Justification: The imaging data acquired during the pre-night test run were included in the model outlined in the pre-registration. However, the corresponding contrasts were not considered of interest as the number of volumes acquired during the three blocks of practice (for each sequence) was lower than expected (participant reached a high level of performance at the end of training which reduced time on task / number of volumes acquired for this session). We optimized the number of blocks in this pre- night test run in order to provide a reflection of end-of-training behavioral performance after allowing for the dissipation of fatigue effects (see [2]), which made these runs less suitable for imaging analyses, especially when performance reaches high levels.</td></tr>
</table>

10. Participant characteristics

Table S5. **Participants' characteristics, sleep and vigilance**. Group averaged (+/- 95% confidence interval) sleep characteristics leading up to the experimental session and vigilance assessments at time of testing (N=31 if not specified otherwise)

**Number of datasets per analysis (number of female)**				
Behavior & fMRI		28 (14)		
EEG and polysomnography		30 (15)		
Closed-loop stimulation		31(15)		
Complete datasets		27 (14)		
**Participant characteristics**				
Age (yrs)		23.7 ranging from 18 to 30		
Edinburgh Handedness [3]		89.0 [84.1 - 95.9]		
Stanford Sleepiness Scale [4]		6.0 [4.8 – 7.2]		
Beck Depression Scale [5]		3.1 [1.9 - 4.3]		
Beck Anxiety Scale [6]		2.6 [1.6 – 3.7]		
PSQI [7]		2.6 [2.1 - 3.1]		
Chronoscore (CRQ)[8]		53.1 [50.3 - 55.1]		
**Sleep duration** [a] (N = 30)				
Night 1 (min)		485.5 [471.3 - 499.6]		
Night 2 (min)		492.9 [477.9 - 507.9]		
Night 3 (min)		499.7 [486.6 - 512.8]		
**St. Mary's questionnaire**		*Quality*	*Duration (min)*	
Night 3		4.1 [3.9 - 4.4]	480.3 [463.2 - 497.4]	
Experimental Night		3.7 [3.4 – 4.0]	435.4 [422.8 – 448.0]	
One-way rmANOVA results		$F(1,30) = 5.18$; $p = 0.03$	$F(1,30) = 19.09$; $p < 0.001$	
**Psychomotor Vigilance Task** [b] (N = 26)				
Pre-night RT (ms)		310.1 [306.4 – 313.7]		
Post-night RT (ms)		314.5 [311.2 – 317.8]		
One-way rmANOVA results		$F(1,25) = 0.70$; $p = 0.42$		
**Stanford sleepiness score**				
Pre-night Session		2.2 [1.9 - 2.4]		
Post-night Session		2.2 [1.9 - 2.5]		
One-way rmANOVA results		$F(1,30) = 0.18$; $p = 0.68$		
**Sleep characteristics** (N = 30)				
Time allowed to sleep (min)		450 [436.8 – 463.1]		
Total Sleep Time [c] (min)		374.5 [354.9 - 354.9]		
Sleep Efficiency [d] (%)		83.3 [79.6 - 87]		
NREM1 Latency (min)		13.7 [17.7 - 9.7]		
Time awake (min)		39.7 [25.9 - 53.5]		
Time in NREM1 Sleep (min)		34.9 [28.9 - 41.0]		
Time in NREM2 Sleep (min)		229.4 [216.6 - 242.1]		
Time in NREM3 Sleep (min)		74.5 [63.3 - 85.7]		
Time in REM Sleep (min)		70.6 [61.8 - 79.4]		
**Participants reaching** (N = 30)				
NREM3 sleep		30		
REM sleep		30		
**Algorithm accuracy** [e] (N = 31)		*All cues*	*Up-reactivated cues*	*Down-reactivated cues*
True positive	Number	1223.5 [979.9 – 1467.1]	622.9 [495.2 – 750.7]	600.5 [483.3 - 717.7]
	Percentage	82.2 % [79.6 – 84.8]	89.5 % [87.8 - 91.2]	74.9 % [71.7 - 78.1]
**Number of Auditory cues** (N = 30)		*All cues*	*Up-reactivated cues*	*Down-reactivated cues*
During all stages		1492.2 [1228.0 -1756.5]	700.6 [566.0 – 835.1]	791.7 [660.1 – 923.2]
During wake		8.3 [4.6 - 11.9]	2.7 [1.5 – 4.0]	5.5 [2.1 - 8.9]
During NREM1 Sleep		6.3 [3.2 - 9.3]	2.5 [0.9 - 4.1]	3.7 [1.7 - 5.8]
During NREM2-3 Sleep		1476.5 [1211.2 - 1741.8]	694.9 [560.0 - 829.9]	781.6 [649.5 - 913.7]
During REM Sleep		1.2 [0.5 – 1.9]	0.4 [0.1 - 0.7]	0.8 [0.3 - 1.4]
Accuracy [f]		98.5 [97.6 - 99.3]	98.8 [98.1 - 99.4]	98.2 [97.2 - 99.3]

Notes. Values are means [lower and upper limit of the 95% Confidence Interval - CI]. PSQI = Pittsburgh Sleep Quality Index; CRQ = Circadian Rhythm Questionnaire. REM: Rapid Eye Movement. [a] Sleep duration was computed as the mean across the actigraphy data and the sleep diary for the three nights before the experimental day (except for three participants for which only the sleep diary data were available, both actigraphy data and sleep diary were missing for one participant). [b] Median of reaction times (RT) computed across the 100 trials of each session. REM: Rapid Eye Movement. [c] Total sleep time was computed as the total time spent in stages NREM2-3 and REM sleep. [d] Sleep efficiency was computed as the percent of time asleep (namely in NREM2-3 and in REM sleep) relative to the total time in bed (specifically, from lights off to lights on). [e] Algorithm accuracy was based on the offline SO detection performed on Fpz which was recorded for all 31 participants. The number of cues reported in this Table is slightly different than the number of analyzed events reported in the main text (see Methods) due to the preprocessing methods for artefact rejection. [f] Percentage of auditory cues correctly sent during NREM2 and NREM3 sleep based on the PSG scoring available for 30 participants.

11. Negative control analyses

Impact of the number of cues on offline changes in performance

Exploratory correlation analyses were performed to test the relationship between the offline changes in performance and the total number of cues in each condition. No significant correlations were observed for any of the conditions (up-stimulation: r(25) = 0.014, p = 0.55; down-stimulation: r(25) = 0.00018, p = 0.95). Similar results were observed for the correlation between offline changes in performance and the number of true positive cues only (up-stimulation: r(25) = 0.014, p = 0.55; down-stimulation: r(25) = 0.00017, p = 0.95).

Equivalent number of discarded trials in the different sessions and conditions

The mean percent of trials discarded from the analyses (i.e., incorrect and outlier trials) is presented in Table S6 for the different sessions and conditions. We tested whether the number of excluded trials was different between conditions in the blocks of practice used to (1) perform the MRI contrasts (i.e., pre-night training vs. post-night training blocks) and (2) compute offline changes in performance (i.e., pre-night test blocks vs. first 3 blocks of post-night training). The rmANOVA on the percentage of discarded trials (incorrect and outlier) with condition (up- vs. down- vs. not-reactivated) and MRI sessions (pre-night training vs. post-night training) as within-subject factors revealed no significant main effects nor any interaction (Session effect: F (1,27) = 0.83, p = 0.37, η^2 = 0.0.030; Condition effect: F (2,54) = 0.37, p = 0.70 (0.69 sphericity corrected), η^2 = 0.013; session by condition interaction: F (2,54) = 1.81, p = 0.17 (0.18 sphericity corrected), η^2 = 0.063). The rmANOVA performed on the percentage of discarded trials with condition and "offline computation" session (pre-night test vs. 3 first blocks of post-night training) as within-subject factors revealed no significant main effects nor any interaction (Session effect: F(1,27) = 0.097, p = 0.76, η^2 = 0.0036; Condition effect: F(2,54) = 0.83, p = 0.44 (0.43 sphericity corrected), η^2 = 0. 0.030; session by condition interaction: F (2,54) = 0.57, p = 0.57 (0.55 sphericity corrected), η^2 = 0.021). These analyses confirm that the number of analyzed trials did not differ among conditions.

Table S6: Mean percent of discarded trials in the behavioral analysis and 95% confidence interval.

	Pre-night training	Pre-night testing	Post-night (3 first blocks)	Post-night training (all blocks)
Up-reactivated	10.9 [7.9 - 13.9]	9.6 [6.3 - 13]	9.9 [6.7 - 13.1]	9.9 [7.4 - 12.4]
Down-reactivated	10.9 [8.8 - 13.1]	12.6 [8.7 - 16.4]	11 [8.3 - 13.6]	11.4 [9.4 - 13.3]
Not-reactivated	10.5 [8.5 - 12.5]	9.8 [7.7 - 11.8]	10.1 [6.9 - 13.3]	9.5 [7.5 - 11.6]

Equivalent vigilance during each behavioral session

As presented in supplementary Table S5, the one-way rmANOVAs on both the median reaction times of the PVT and the Stanford Sleepiness Scale score with Session as two-level factor (pre-night and post-night) showed no significant main effect of session (PVT: F(1,25) = 0.7; p = 0.42; SSS: F(1,30) = 0.18; p = 0.68), suggesting that vigilance did not differ between the two testing sessions.

Equivalent baseline performance between the three movement sequences

We assessed whether performance (speed and accuracy) during initial training significantly differed between the three movement sequences irrespective of the reactivation condition. To do so, two two-way rmANOVAs with sequence (A vs. B vs. C) and Block (1st rmANOVA on the 21 blocks of the pre-night training and 2nd rmANOVA on the 3 blocks of the pre-night test) as within-subject factors were performed on the sequential SRRT performance (Figure S7).

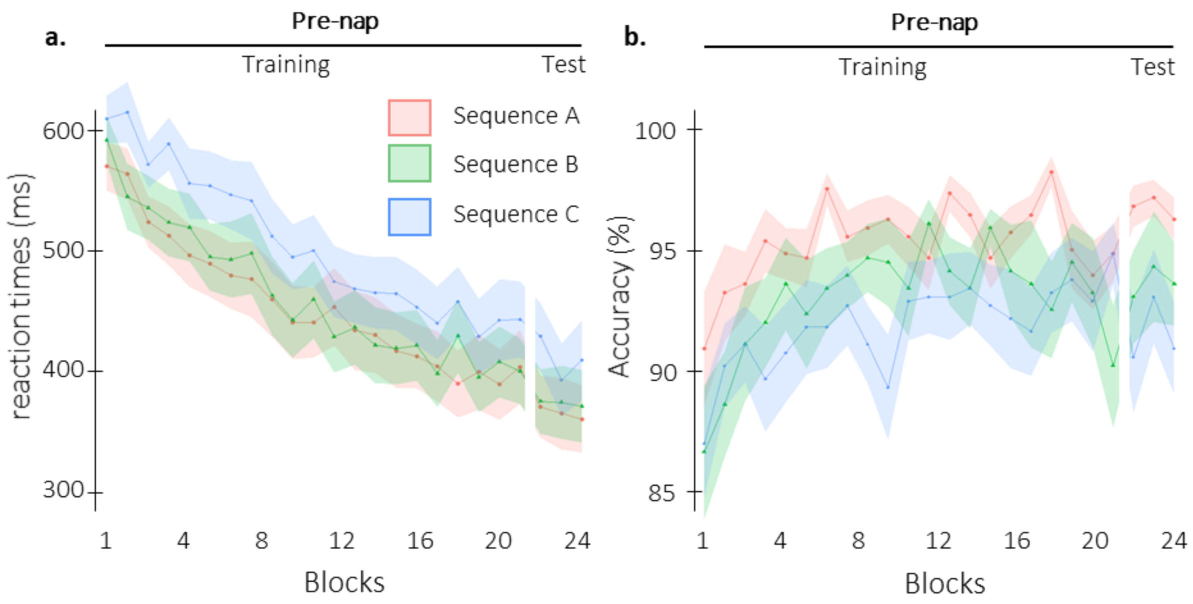

Figure S7: Behavioral results per sequence *(n = 28)*. ***a. Performance speed*** *(mean reaction time in ms +/- standard error in shaded regions) across participants plotted as a function of blocks of practice during the pre-night session (Training and Test) for the sequence A (4 7 2 8 3; red), the sequence B (1 6 3 5 2; green), and the sequence C (7 3 8 4 6; blue). Analyses of the pre-night training data (repeated-measures ANOVA using sequences (3) and blocks (20) as within-subject factors) indicated differences in movement speed among the 3 sequences (main effect of Sequence (F(2,54) = 20.72, p-value < 0.001, η² = 0.43), a result that can be attributed to slower response times for Sequence C. However, performance improved similarly among sequences (main effect of Block: F(20,540) = 42.5, p-value < 0.001, η² = 0.61; Block by Sequence interaction: F(40,1080)= 1.19, p-value = 0.20, η² = 0.04). Analyses of the pre-night test data (repeated-measures ANOVA using sequences (3) and blocks (4) as within-subject factors)showed that sequence C was still performed significantly slower than the others sequence (main effect of Sequence (F(2,54) = 20.72, p-value < 0.001 (< 0.001 sphericity corrected), η² = 0.30), yet performance remained similarly stable across blocks among sequences (main effect of Block: F(20,54) = 1.62, p-value = 0.21 (0.21 sphericity corrected), η² = 0.057; Block by Sequence interaction: F(4,108)= 1.45, p-value = 0.22(0.22 sphericity corrected), η² = 0.051).* ***b. Performance accuracy*** *(mean percentage of correct responses +/- standard error in shaded regions) across participants plotted as a function of blocks of practice during the pre-night session (Training and Test) for sequences A, B and C. Analyses of the pre-night training data (same model as above) indicate a similar pattern as for speed with a main effect of Sequence (F(2,54) = 5.9, p-value = 0.0048, η² = 0.18), a result that can be attributed to better performance for Sequence C. However, accuracy improved similarly among sequences (F(20,540) = 3.0, p-value < 0.001, η² = 0.10; Block by Sequence interaction (F(40,1080)= 1.2, p-value = 0.18, η² = 0.04). Analyses of the pre-night test data (same model as above) showed a similar pattern with a main effect of Sequence (F(2,54) = 6.59, p-value = 0.0027 (0. 0027 sphericity corrected), η² = 0.20), yet no effect of Block (F(2,54) = 0.81, p-value = 0.45 (0.44 sphericity corrected), η² = 0.029) nor interaction of Block by Sequence were observed (F(4,108)= 0.24, p-value = 0.92 (0.89 sphericity corrected), η² = 0.0088). Collectively, these results indicate differences in performance between sequences but as sequence-condition combinations were pseudo-randomized, it is unlikely that this affected the results presented in the manuscript (and see below for negative control analyses on conditions).*

Equivalent baseline performance between the three conditions

Similar analyses testing for baseline differences between the reactivated-up, the reactivated-down, and the non-reactivated sequences were performed.

Analyses of the pre-night training data indicated a significant effect of block, but no main effect of condition or block by condition interaction suggesting that participants learned the three sequence conditions to a similar extent during initial learning (21 blocks of training; main effect of Block: F(20,540) = 42.5, p-value < 0.001, η² = 0.61; main effect of Condition: F(2,54)= 0.53; p-value = 0.59, η² = 0.02; Block by Condition interaction: F(40,1080)= 1.14, p-value = 0.26, η² = 0.04; Figure 2). Similar results were observed for accuracy, i.e., a significant main effect of Block (F(20,540) = 3.0, p-value < 0.001, η² = 0.10)

but no main effect of Condition (F(2,54) = 0.087, p-value = 0.92, η^2 = 0.0032) nor Block by Condition interaction (F(40,1080)= 1.05, p-value = 0.38, η^2 = 0.037, Figure S9a).

Analyses of the pre-night test data showed no main effect of block, condition or block by condition interaction (main effect of Block: F(2,54) = 1.62; p-value = 0.21, η^2 = 0.057; main effect of Condition: F(2,54) = 0.23; p-value = 0.80, η^2 = 0.0084; Block by Condition interaction: F(3,69)= 1.21; p-value = 0.31, η^2 = 0.0063; Figure 2) suggesting that asymptotic performance was reached for all three conditions in the test session that followed the initial training run. Similar results were observed for performance accuracy (main effect of Block: F(2,54) = 0.81; p-value = 0.45, η^2 = 0.029; main effect of Condition: F(2,54) = 0.34; p-value = 0.69, η^2 = 0.99; Block by Condition interaction: F(3,69)= 0.20; p-value = 0.94, η^2 = 0.0075; Figure S9a).

Equivalent impact of the three different sounds

To test the hypothesis of a differential impact of the sounds on motor memory, we ran the same rmANOVA as in our main behavioural analysis but with Sound type (low-frequency tone vs. white noise vs. high-frequency tone) instead of Condition (up- vs. down- vs. not-reactivated) as within-subject factor. We found no significant main effect of the Sound type factor on offline changes in performance (F(2,54) = 30.82, p = 0.48 (0.43 sphericity corrected), η^2 = 0.029; Figure S8).

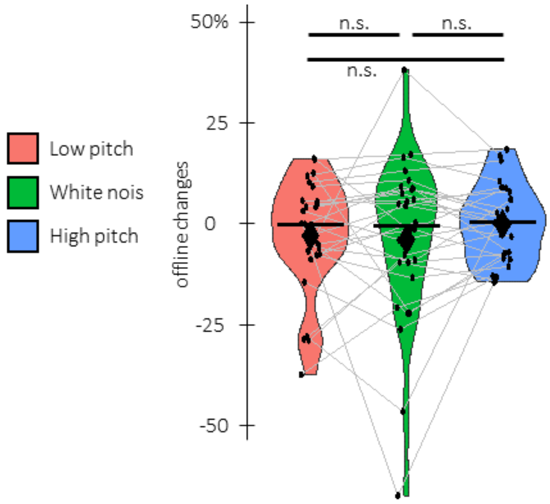

Figure S8: Offline changes in performance speed as a function of the sound attributed to the sequence *(n = 28). (% change between the average of the three blocks of pre-night test and the first three blocks of post-night training) averaged across participants for the low pitch (red), the white noise (green), and the high pitch (blue) sounds. The rmANOVA using Sound type (low-frequency tone vs. white noise vs. high-frequency tone) as within-subject factor showed no significant main effect of the Sound type on offline changes in performance (F(2,54) = 30.82, p = 0.48 (0.43 sphericity corrected),* η^2 *= 0.029). Violin plots: median (horizontal bar), mean (diamond), the shape of the violin plots depicts the kernel density estimate of the data. Black points represent individual data, jittered in arbitrary distances on the x-axis within the respective violin plot to increase perceptibility. For each individual, performance on the different sound conditions are connected with a line between violin plots. n.s: non-significant (rmANOVA).*

To test the hypothesis of a differential impact of the sounds on sleep oscillations, we ran the same analysis as the main EEG analysis with Sound type (low-frequency tone vs. white noise vs. high-frequency tone) as within-subject factor. We did not find any significant positive or negative clusters neither in the SO amplitude nor in the oscillatory analyses comparing the three different sounds (all p-values > 0.070).

Motor execution performance

We computed the *overall performance change* for both the sequential SRTT (first 4 blocks of the pre-night training vs. 4 last blocks of post-night training collapsed across sequences) and the pseudo-random version of the SRTT (4 blocks pre-night session vs. 4 blocks post-night session).

Two-tailed paired Student t-test revealed that overall changes in performance were significantly higher for the sequential SRTT as compared to the random SRTT (t = -10.2, df = 27, p-value < 0.001; Cohen's d = 1.92). Thus, the response time decrease reported on the sequential SRTT in the result section reflects motor sequence learning rather than a mere improvement in motor execution.

12. Analyses on accuracy

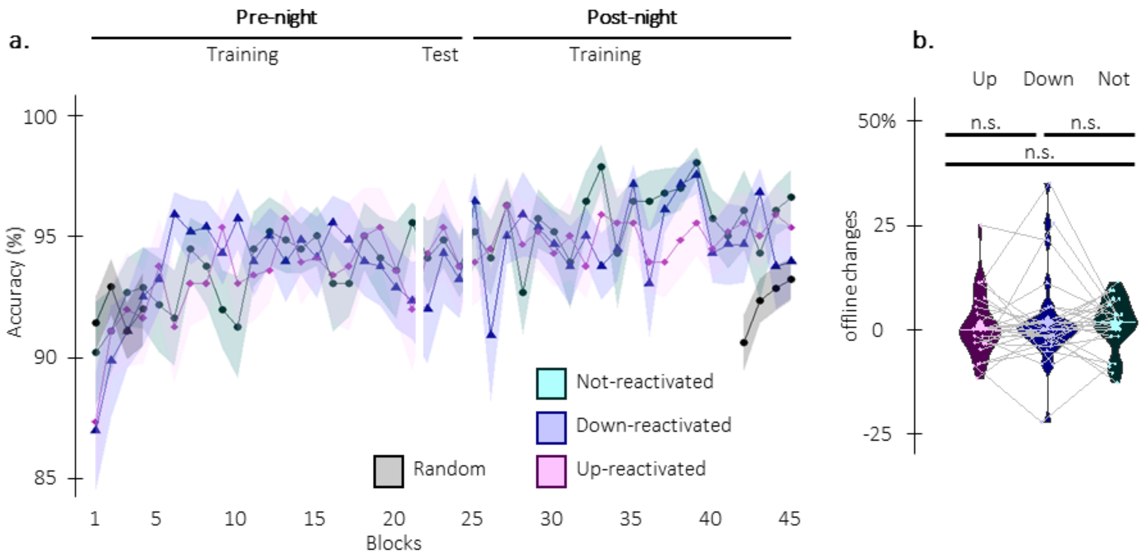

*Figure S9: **Behavioral results on accuracy data** (n = 28). **a. Performance accuracy** (mean percentage of* correct responses*) across participants plotted as a function of blocks of practice during the pre and post-night sessions (+/- standard error) for the up-reactivated (magenta), the down-reactivated (blue) and the not-reactivated (green) sequences. Repeated-measures ANOVAs on the Pre-night data using condition (3) and blocks (20 for Training and 4 for test) as within-subject factors showed a significant main effect of Block during training (F(20,540) = 3.0, p-value < 0.001, η^2 = 0.10) that was no longer significant during test (F(2,54) = 0.81; p-value = 0.45, η^2 = 0.029). There was no main effect of Condition (training: F(2,54) = 0.087, p-value = 0.92, η^2 = 0.0032; test: F(2,54) = 0.34; p-value = 0.69, η^2 = 0.99) nor Block by Condition interaction (training: F(40,1080)= 1.05, p-value = 0.38, η^2 = 0.037; test: F(3,69)= 0.20; p-value = 0.94, η^2 = 0.0075). **b. Offline changes in performance accuracy** (% change between the average of the three blocks of pre-night test and the first three blocks of post-night training) averaged across participants. There was no main effect of condition. A one-way repeated measure analysis of variance (rmANOVA) performed on offline changes in performance accuracy with Condition (reactivated-up vs. reactivated-down vs. non-reactivated) as within-subject factor showed no significant Condition effect (F (2,54) = 0.12, p = 0.89 (0.87 sphericity corrected), η^2 = 0.0043). Violin plots: median (horizontal bar), mean (diamond), the shape of the violin plots depicts the distribution of the data. Colored points represent individual data, jittered in arbitrary distances on the x-axis within the respective violin plot to increase perceptibility. For each individual, performance on the different conditions is connected with a line between violin plots. n.s: non-significant.*

13. All NREM stimulation analysis

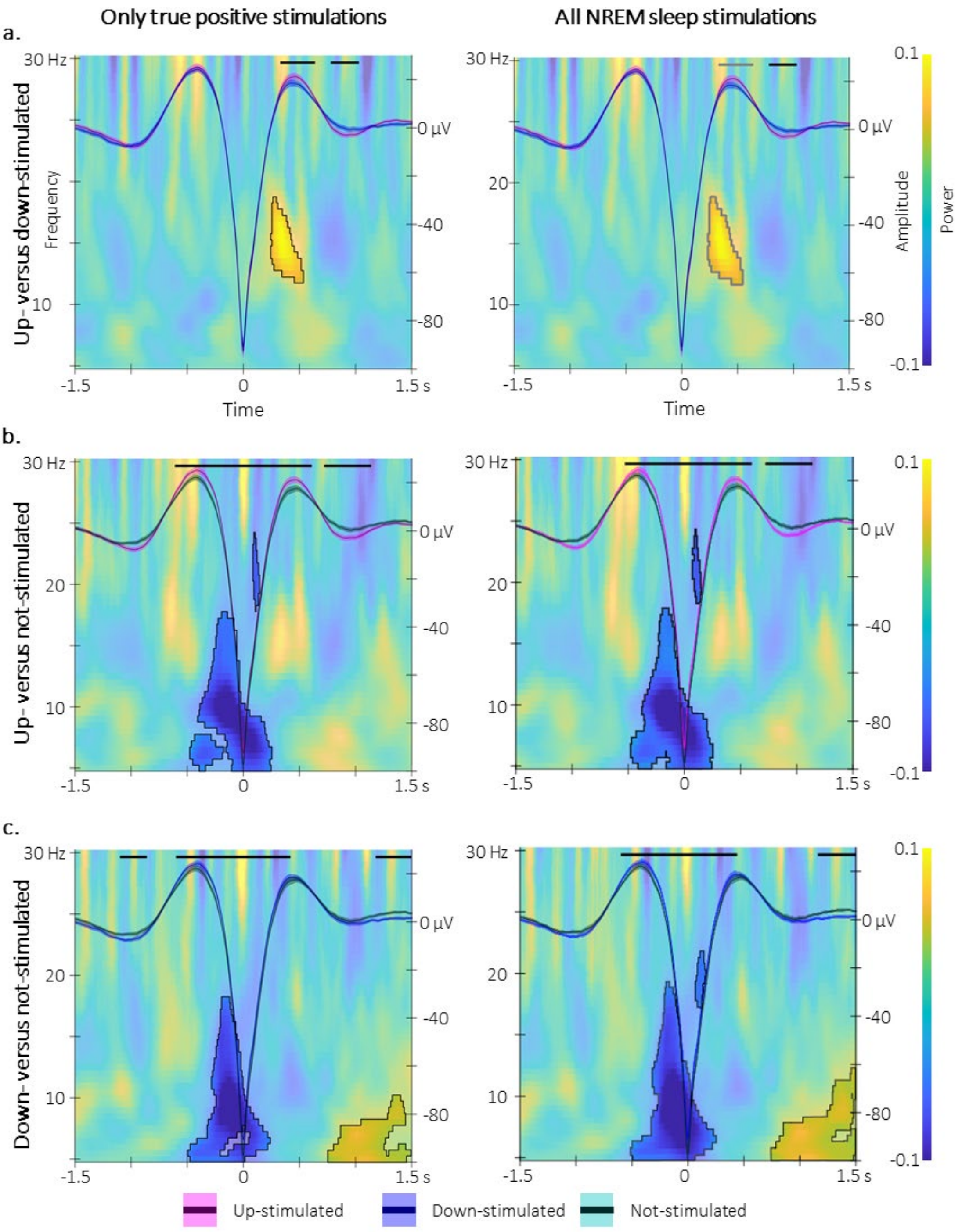

Figure S10: Electrophysiological results of the analyses including only the true positive stimulation (left column and Figure 3 of the main manuscript) and including all the NREM2-3 sleep stimulations (right column) *(n = 30)*. *Statistical comparisons were performed using non-parametric cluster-based permutations.* ***a. Up- vs down-stimulated contrasts****. Time-frequency representations (TFR) of the difference in power modulation illustrated at Cz around the trough of the up- and down-stimulated SOs on which the grand-average of the SOs illustrated on Fz is super-imposed (magenta(blue): up(down)-stimulated SO). Horizontal lines represent the adjacent time points of the significant spatio-temporal clusters showing a difference in SO amplitude between the two trough-locked ERPs (horizontal grey lines show cluster significant after permutations [cluster p-value < 0.05] and horizontal black lines after permutation and correction for multiple comparisons [cluster p-value < 0.0083]). Results show that the up-stimulated SO presented greater amplitude at their peak (****left****: 0.33 to 0.64 sec post-trough;* ***right****: 0.47 to 0.62 sec post-trough) followed by a deeper deflection (****left****: from 0.78 to 1.03 sec post-trough;* ***right****: from 0.78 to 1.02 sec post-trough). Further, the area highlighted in the TFR represents the adjacent time-frequency points of the significant spatio-temporal-frequency cluster showing a difference in power between conditions (grey contour shows cluster significant after permutations [cluster p-value < 0.05] and black contour after permutation and correction for multiple comparisons [cluster p-value < 0.0083]). Sigma power nested in the ascending phase of the up-stimulated SOs was greater than for the down-stimulated SOs (****left****: from 0.25 to 0.4 sec post-trough and from 12 to 17 Hz;* ***right****: from 0.25 to 0.37 sec post-trough and from 13.5 to 19 Hz).* ***b. Up- vs not-stimulated contrasts****. Same as panel a but for the up- and the not-stimulated trough-locked SO potential (magenta and green, respectively) and power modulation. Results show that the amplitude of the up-stimulated SOs was greater than the not-stimulated SOs from -0.61 to 0.61 sec (****left****) / from -0.53 to 0.60 sec (****right****) while it reversed from 0.72 to 1.14 sec (****left****) / from 0.72 to 1.14 sec (****right****) relative to the trough onset. Power in the 5-17.5 Hz (****left****) / 5-19 Hz (****right****) frequency range was lower in the up- as compared to the not-stimulated condition in the descending phase of the SOs (****left****: from -0.45 to 0.24 sec relative to the SO trough;* ***right****: from -0.48 to 0.27 sec relative to the SO trough).* ***c. Down- vs not-stimulated contrasts****. Same as panel a but for the down- and the not-stimulated trough-locked SO potential (blue and green, respectively) and power modulation. Results show that the amplitude of the down-stimulated SOs was greater than the not-stimulated SOs from -0.60 to 0.42 sec (****left****) / from -0.60 to 0.44 sec (****right****) while it reversed from 1.16 to 1.50 sec (****left****) / (****right****) relative to the trough onset. Power was lower in the 5-18 Hz (****left****) / 5-19 Hz and 17-22 Hz (****right****) frequency range in the down- as compared to the not-stimulated condition during the descending phase of SOs (****left****: from -0.49 to 0.24 sec relative to the SO trough;* ***right****: from -0.52 to 0.27 sec relative to the SO trough) and greater in the 5-10 Hz from 0.76 to 1.50 sec (****left****) / 5-12 Hz from 0.70 to 1.50 sec (****right****) relative to trough onset.*

14. Seed regions for connectivity analyses

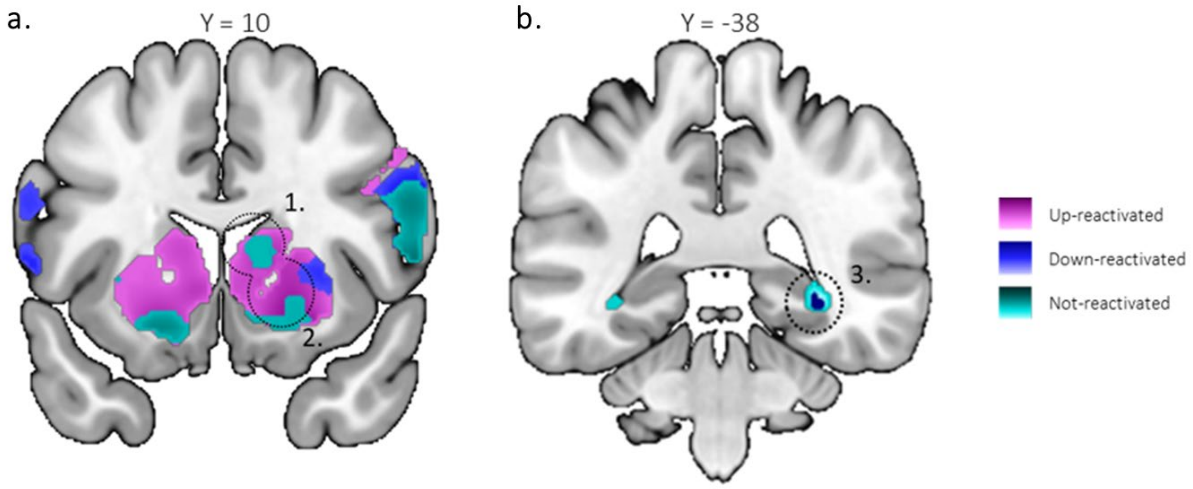

Figure S11: Seed regions for the connectivity analyses (n = 28). *Task-related functional connectivity was examined using psychophysiological interaction analyses. Connectivity of three seed regions revealed by the activation-based analyses was assessed.* ***a. Striatal seeds (left panel).*** *(1) right caudate (x = 10, y = 14, z = 12) and (2) right putamen (x = 18, y = 12, z = -2).* ***b. Hippocampal seed (right panel)*** *(3) right hippocampus (x = 32, y = -38, z = -6). For each individual, the first eigenvariate of the signal was extracted using Singular Value Decomposition of the time series across the voxels included in a 10 mm-radius sphere centered on these coordinates and shown with the dotted circles. Seed spheres are displayed on a T1-weighted template image as part of the MRIcroGL software (https://www.mccauslandcenter.sc.edu/mricrogl) with a threshold of p < 0.005 uncorrected.*

15. Functional role of the brain regions selected *a priori* for the MRI analyses

Our statistical analyses were performed across all the voxels of a large mask including a set of task-relevant brain regions known to play a critical role in motor sequence learning [10–13] and consisting of M1, PMC, SMA, aSPL, striatum and hippocampus. Note that this motor-network mask approach was chosen instead of a whole-brain approach to limit the number of identified clusters to *a priori*-defined task-relevant brain regions.

There is extensive evidence that motor sequence learning is associated with modulation of BOLD signal in these brain regions, [14–18]. These different models converge toward the general idea that parallel circuits operate during motor sequence learning to support the development of different features of the motor sequences. The learning of the spatial coordinates of the task recruits hippocampo-parietal circuits as well associative striatal areas whereas learning of the motor coordinates relies on M1-sensorimotor-striatum networks. Evidence reviewed in these models suggest that the transformation between these two coordinate systems is mediated by the SMA and the premotor cortex. Importantly, recent neuroimaging work has provided greater insight into the type of information represented in these brain regions (e.g., finger vs. sequence representation in M1, sequence vs. time representation in the PMC, movement-position binding in the striatum, response-goal-position binding in hippocampo-parietal networks)[11,12,19]. Importantly, these regions have also been shown to play a critical role in the (wake and sleep-related) consolidation process [20] REF. Specifically, sleep differently influences brain activity depending on the nature of the information supported by these different brain regions. Abstract (spatial) representations encoded in hippocampo-cortical regions have been shown to require sleep to consolidation while the consolidation of motoric representations is thought to occur over both wake and sleep intervals. It is proposed that the between-representation transformation maps developed during learning in the SMA and PMC undergo sleep-dependent consolidation and facilitate the interaction between the spatial and motor representations of learning over sleep intervals to optimize motor behavior [20]. Based on the literature reviewed above, we expected that up- (as compared to both down- and not-) stimulation would specifically result in strengthening task-related brain activity (at retest as compared to training) in hippocampo-parietal networks. We also expected that activity in striato-motor networks would increase after the consolidation period in concert with (e.g., via functional interaction with) hippocampal-cortical networks for the up condition as compared to both the down and not conditions

16. Coordinates for small volume corrections

Area	x mm	y mm	z mm	Reference
HIPPOCAMPUS				
	26	-34	-6	Albouy et al., 2008[21]
	30	-24	-15	Schendan et al., 2003[22]
	24	-33	-9	Schendan et al., 2003[22]
	-18	-16	-14	Strange et al., 1999[23]
	24	-34	-2	Strange et al., 1999[23]
	38	-34	-2	Albouy et al., 2008[21]
	34	-38	-8	Albouy et al., 2008[21]
	-18	-34	4	Albouy et al., 2008[21]
STRIATUM				
Putamen	±28	-14	-8	Albouy et al., 2008[21]
	29	-6	5	Lehericy et al., 2005[24]
	27	6	-6	Schendan et al., 2003[22]
	24	10	-8	Van Der Graaf et al., 2004[25]
Caudate	20	-2	26	Albouy et al., 2008[21]
	18	8	20	Dolfen et al., 2021[26]
	10	26	4	Gann et al., 2021[27]
	15	12	12	Schendan et al., 2003[22]
	18	22	12	Albouy et al., 2008[21]
MOTOR REGIONS				
aSPL	±46	-38	38	Gann et al., 2021[27]
	±46	-46	46	Penhune & Doyon 2005[28]
M1	[33 – 39]	[18 – 27]	[51 – 63]	Lehericy et al., 2006[29]
	[21 – 40]	[-6 – 11]	[44 – 51]	Paus, 1996[30]
	50	-18	42	Penhune & Doyon 2005[28]
PMC	50	-12	46	Penhune & Doyon 2005[28]

Table S7: Coordinates of areas of interest used for spherical small volume corrections for the results presented in the main text.

Table S8: Description of the published works used for spherical small volume corrections for the results presented in the main text. SRTT: Serial Reaction Time Task; FTT: Finger Taping Task.

Reference	Cognitive process	Memory Type	Awareness of learned material	Task
Albouy et al., 2008[21]	Learning and consolidation	Motor	Implicit	Oculomotor SRTT
Schendan et al., 2003[22]	Learning	Motor	Implicit and explicit	SRTT
Strange et al., 1999[23]	Learning	Episodic	Implicit	Artificial grammar learning
Lehericy et al., 2005[24]	Learning	Motor	Explicit	FTT
Van Der Graaf et al., 2004[25]	Learning	Motor	Implicit	SRTT
Dolfen et al., 2021[26]	Learning and consolidation	Motor	Explicit	FTT
Gann et al., 2021[27]	Learning	Motor	Implicit	SRTT
Penhune & Doyon, 2005[28]	Learning and consolidation	Motor	Explicit	FTT
Lehericy et al., 2006[29]	Learning	Motor	Explicit	FTT
Paus, 1996[30]	Learning	Motor	Implicit	Oculomotor

References

1. Combrisson, E. *et al.* Tensorpac: An open-source Python toolbox for tensor-based phase-amplitude coupling measurement in electrophysiological brain signals. *PLoS Comput Biol* **16**, e1008302 (2020).
2. Pan, S. C. & Rickard, T. C. Sleep and motor learning: Is there room for consolidation? *Psychological Bulletin* **141**, 812–834 (2015).
3. Oldfield, R. C. The assessment and analysis of handedness: The Edinburgh inventory. *Neuropsychologia* **9**, 97–113 (1971).
4. Hoddes, E., Dement, W. & Zarcone, V. *Development and Use of Stanford Sleepiness Scale (SSS)*. vol. 9 (1972).
5. Beck, A. T., Steer, R. A., Ball, R. & Ranieri, W. F. Comparison of Beck Depression Inventories-IA and-II in Psychiatric Outpatients. *Journal of Personality Assessment* **67**, 588–597 (1996).
6. Beck, A. T., Epstein, N., Brown, G. & Steer, R. A. An inventory for measuring clinical anxiety: Psychometric properties. *Journal of Consulting and Clinical Psychology* **56**, 893–897 (1988).
7. Buysse, D. J., Reynolds, C. F., Monk, T. H., Berman, S. R. & Kupfer, D. J. The Pittsburgh sleep quality index: A new instrument for psychiatric practice and research. *Psychiatry Research* **28**, 193–213 (1989).
8. Horne, J. A. & Ostberg, O. A self-assessment questionnaire to determine morningness-eveningness in human circadian rhythms. *Int J Chronobiol* **4**, 97–110 (1976).
9. Ngo, H.-V. V. & Staresina, B. P. Shaping overnight consolidation via slow-oscillation closed-loop targeted memory reactivation. *Proc. Natl. Acad. Sci. U.S.A.* **119**, e2123428119 (2022).
10. Dolfen, N., Reverberi, S., Op De Beeck, H., King, B. R. & Albouy, G. The hippocampus binds movements to their temporal position in a motor sequence. Preprint at https://doi.org/10.1101/2022.12.20.521084 (2022).
11. Berlot, E., Popp, N. J. & Diedrichsen, J. A critical re-evaluation of fMRI signatures of motor sequence learning. *eLife* **9**, e55241 (2020).

12. Yokoi, A. & Diedrichsen, J. Neural Organization of Hierarchical Motor Sequence Representations in the Human Neocortex. *Neuron* **103**, 1178-1190.e7 (2019).
13. Yokoi, A., Arbuckle, S. A. & Diedrichsen, J. The Role of Human Primary Motor Cortex in the Production of Skilled Finger Sequences. *J Neurosci* **38**, 1430–1442 (2018).
14. Albouy, G., King, B. R., Maquet, P. & Doyon, J. Hippocampus and striatum: Dynamics and interaction during acquisition and sleep-related motor sequence memory consolidation: Hippocampus and Striatum and Procedural Memory Consolidation. *Hippocampus* **23**, 985–1004 (2013).
15. Dayan, E. & Cohen, L. G. Neuroplasticity Subserving Motor Skill Learning. *Neuron* **72**, 443–454 (2011).
16. Doyon, J., Penhune, V. & Ungerleider, L. G. Distinct contribution of the cortico-striatal and cortico-cerebellar systems to motor skill learning. *Neuropsychologia* **41**, 252–262 (2003).
17. Hikosaka, O., Nakamura, K., Sakai, K. & Nakahara, H. Central mechanisms of motor skill learning. *Current Opinion in Neurobiology* **12**, 217–222 (2002).
18. Penhune, V. B. & Steele, C. J. Parallel contributions of cerebellar, striatal and M1 mechanisms to motor sequence learning. *Behavioural Brain Research* **226**, 579–591 (2012).
19. Dolfen, N., Reverberi, S., Op De Beeck, H., King, B. R. & Albouy, G. The hippocampus represents information about movements in their temporal position in a learned motor sequence. *J. Neurosci.* e0584242024 (2024) doi:10.1523/JNEUROSCI.0584-24.2024.
20. King, B. R., Hoedlmoser, K., Hirschauer, F., Dolfen, N. & Albouy, G. Sleeping on the motor engram: The multifaceted nature of sleep-related motor memory consolidation. *Neuroscience & Biobehavioral Reviews* **80**, 1–22 (2017).
21. Albouy, G. *et al.* Both the Hippocampus and Striatum Are Involved in Consolidation of Motor Sequence Memory. *Neuron* **58**, 261–272 (2008).
22. Schendan, H. E., Searl, M. M., Melrose, R. J. & Stern, C. E. An fMRI Study of the Role of the Medial Temporal Lobe in Implicit and Explicit Sequence Learning. *Neuron* **37**, 1013–1025 (2003).
23. Strange, B. A., Fletcher, P. C., Henson, R. N. A., Friston, K. J. & Dolan, R. J. Segregating the functions of human hippocampus. *Proc. Natl. Acad. Sci. U.S.A.* **96**, 4034–4039 (1999).

24. Lehéricy, S. *et al.* Distinct basal ganglia territories are engaged in early and advanced motor sequence learning. *Proc. Natl. Acad. Sci. U.S.A.* **102**, 12566–12571 (2005).

25. Van Der Graaf, F. H. C. E., De Jong, B. M., Maguire, R. P., Meiners, L. C. & Leenders, K. L. Cerebral activation related to skills practice in a double serial reaction time task: striatal involvement in random-order sequence learning. *Cognitive Brain Research* **20**, 120–131 (2004).

26. Dolfen, N. *et al.* Stress Modulates the Balance between Hippocampal and Motor Networks during Motor Memory Processing. *Cerebral Cortex* **31**, 1365–1382 (2021).

27. Gann, M. A. *et al.* Hippocampal and striatal responses during motor learning are modulated by prefrontal cortex stimulation. *NeuroImage* **237**, 118158 (2021).

28. Penhune, V. B. & Doyon, J. Dynamic Cortical and Subcortical Networks in Learning and Delayed Recall of Timed Motor Sequences. *J. Neurosci.* **22**, 1397–1406 (2002).

29. Lehéricy, S. *et al.* Motor control in basal ganglia circuits using fMRI and brain atlas approaches. *Cerebral Cortex* **16**, 149–161 (2006).

30. Paus, T. Location and function of the human frontal eye-field: a selective review. *Neuropsychologia* **34**, 475–483 (1996).
