# Supplementary material for: Unraveling the neurophysiological correlates of phase-specific enhancement of motor memory consolidation via slow-wave closed-loop targeted memory reactivation
Source: Nat Commun. 2025 Mar 18;16:2644. doi: 10.1038/s41467-025-57602-2 (PMC11920436; doi:10.1038/s41467-025-57602-2)
Supplement: Supplementary file 3 — Reporting Summary [file 41467_2025_57602_MOESM3_ESM.pdf]

nature portfolio

Corresponding author(s): Genevieve Albouy

Last updated by author(s): 12/12/2024

Reporting Summary

Nature Portfolio wishes to improve the reproducibility of the work that we publish. This form provides structure for consistency and transparency in reporting. For further information on Nature Portfolio policies, see our Editorial Policies and the Editorial Policy Checklist.

Statistics

For all statistical analyses, confirm that the following items are present in the figure legend, table legend, main text, or Methods section.

n/a	Confirmed	
☐	☒	The exact sample size (n) for each experimental group/condition, given as a discrete number and unit of measurement
☐	☒	A statement on whether measurements were taken from distinct samples or whether the same sample was measured repeatedly
☐	☒	The statistical test(s) used AND whether they are one- or two-sided<br>*Only common tests should be described solely by name; describe more complex techniques in the Methods section.*
☐	☒	A description of all covariates tested
☐	☒	A description of any assumptions or corrections, such as tests of normality and adjustment for multiple comparisons
☐	☒	A full description of the statistical parameters including central tendency (e.g. means) or other basic estimates (e.g. regression coefficient) AND variation (e.g. standard deviation) or associated estimates of uncertainty (e.g. confidence intervals)
☐	☒	For null hypothesis testing, the test statistic (e.g. F, t, r) with confidence intervals, effect sizes, degrees of freedom and P value noted<br>*Give P values as exact values whenever suitable.*
☒	☐	For Bayesian analysis, information on the choice of priors and Markov chain Monte Carlo settings
☒	☐	For hierarchical and complex designs, identification of the appropriate level for tests and full reporting of outcomes
☐	☒	Estimates of effect sizes (e.g. Cohen's d, Pearson's r), indicating how they were calculated

Our web collection on statistics for biologists contains articles on many of the points above.

Software and code

Policy information about availability of computer code

Data collection	Matlab 2019a (Math Works Inc., Natick, MA, USA); Matlab Psychophysics Toolbox version 3; digital sleep recorder (V-Amp, Brain Products, Gilching, Germany; bandwidth: DC to Nyquist frequency); CL-TMR device (Elemind Technologies); Philips Achieva 3.0T MRI system equipped with a 32-channel head coil.
Data analysis	Matlab 2019a (Math Works Inc., Natick, MA, USA); YASA open-source Python toolbox; Matlab fieldtrip toolbox; R: A language and environment for statistical computing RStudio: Integrated Development for R; Statistical parametric mapping (SPM12; Welcome Department of Imaging Neuroscience, London, UK)<br><br>The source code is available at https://github.com/judithnicolas/Closed-Loop-Sleep-Motor-Memory.

For manuscripts utilizing custom algorithms or software that are central to the research but not yet described in published literature, software must be made available to editors and reviewers. We strongly encourage code deposition in a community repository (e.g. GitHub). See the Nature Portfolio guidelines for submitting code & software for further information.

Data

Policy information about availability of data

All manuscripts must include a data availability statement. This statement should provide the following information, where applicable:

- Accession codes, unique identifiers, or web links for publicly available datasets
- A description of any restrictions on data availability
- For clinical datasets or third party data, please ensure that the statement adheres to our policy

The raw data collected in this study have been deposited in the publicnEUro database (https://publicneuro.eu/catalogue.html) under accession code PN000004: Closed Loop Sleep Motor Memory using a brain imaging data structure (BIDS) format. These data are available under restricted access to adhere to the European General Data Protection Regulation. Unlimited access to the data can be provided after signing a Data User Agreement. Users then receive a link to access the data with a 72-h validity. Source data files are provided with this paper.

Research involving human participants, their data, or biological material

Policy information about studies with human participants or human data. See also policy information about sex, gender (identity/presentation), and sexual orientation and race, ethnicity and racism.

Reporting on sex and gender	The study was open to both males and females. Among 31 participants, 15 self-reported having a female biological sex. None reported that their gender does not correspond to their biological sex. EEG analyses parameters (SO detection) were adjusted for sex.
Reporting on race, ethnicity, or other socially relevant groupings	The study was open to individuals from all ethnic and racial backgrounds. Ethnicity was self-reported but was not used in the analyses.
Population characteristics	See below
Recruitment	Participants were recruited via advertisements posted on student job websites or shared on social media. As a result, the majority of the participants were KU Leuven undergraduates students and the recruited sample might therefore not be representative of the more general population.
Ethics oversight	Committee for Medical Ethics of the University Hospitals Leuven (B322201525025)

Note that full information on the approval of the study protocol must also be provided in the manuscript.

Field-specific reporting

Please select the one below that is the best fit for your research. If you are not sure, read the appropriate sections before making your selection.

☐ Life sciences ☒ Behavioural & social sciences ☐ Ecological, evolutionary & environmental sciences

For a reference copy of the document with all sections, see nature.com/documents/nr-reporting-summary-flat.pdf

Behavioural & social sciences study design

All studies must disclose on these points even when the disclosure is negative.

Study description	Intra-individual design with quantitative data (Reaction times, actigraphy data, BOLD activity, and electroencephalogram) and qualitative data (questionnaires, see below and manuscript for details)
Research sample	The study was open to both males and females, as well as to individuals from all ethnic and racial backgrounds. The majority of the participants were KU Leuven undergraduates students. This study examines learning and memory processes in young healthy adults between 18-30 years of age. Children and older adults were therefore not included in this research. Separate, age-specific, studies in the excluded age groups (i.e. children and older adults) are warranted. There is indeed a plethora of research showing that the learning and memory consolidation processes investigated in the current project change across the lifespan. In other words, children, young and older adults do not represent a homogenous cohort for the study of such processes. The investigation of learning and memory changes across the lifespan being beyond the scope of the proposed research, future lifespan-oriented research will address these questions.<br><br>27 complete data sets were analyzed (31 data sets were collected)<br>Inclusion criteria were: 1) right handed; 2) no previous extensive training with a musical instrument or as a professional typist, 3) free of medical, neurological, psychological, or psychiatrie conditions, including depression and anxiety as assessed by the Beck's Depression and Anxiety Inventories, 4) no indications of abnormal sleep, as assessed by the Pittsburgh Sleep Quality Index; 5) not considered extreme morning or evening types, as quantified with the Horne & Ostberg chronotype questionnaire; and, 6) free of psychoactive or sleep-affecting medications. None of the participants were shift-workers or did trans-meridian trips in the last 3 months.
Sampling strategy	The sampling strategy was random.

	We performed a power analysis based on our previous study investigating auditory TMR in an open-loop paradigm (Nicolas et al. 2022). This power analysis was performed with the G*Power software. The partial η^2 was calculated based on our behavioral main effect showing a significant effect of condition (reactivated vs. control) on offline changes in performance speed and was transformed to an effect size f (η^2 = 0.15; f = 0.42). The correlation coefficient calculated between the offline changes in performance speed in the reactivated and the control conditions was 0.66, but due to the different nature of the design (e.g., 3 sequences instead of 2, 5-element sequences instead of 8), we set the average correlation coefficient between repeated measures at r = 0.5 for a more conservative power calculation. Finally, the sphericity correction was set to 0.5 since the primary factors of interest in our design had 3 levels (up-reactivated, down-reactivated and not-reactivated). The primary contrast of interest is a reactivation condition main effect on offline changes in performance speed tested with a one-way rmANOVA. The required sample size for a 95% power is 27 at an alpha error probability of 0.05.
Data collection	The study is a full within-subject design (see Figure 1 in main manuscript). The experimenter was not blind to the randomisation of the conditions. Following a habituation night that was completed approximately one week prior to the experiment, 31 participants (within-subject design) underwent a pre-night motor task session in the scanner, a full night of sleep in the sleep lab monitored with polysomnography during which slow-oscillation closed-loop targeted memory reactivation (CL-TMR) was applied, and a post-night retest session in the scanner. During the motor task (pre-night and post-night sessions), three movement sequences were performed (sequences A, B and C whereby 1 and 8 correspond to the right and left little fingers, respectively) and were cued by three different 100-ms auditory tones. For each movement sequence, the respective auditory tone was presented prior to each sequence execution. Two of these sounds (memory cues) were replayed during the post-learning sleep episode at specific phases of SO (up vs. down, see panel B for details) while the third sound was a control condition, which was not replayed during the night. Note that the sequence / sound / condition combinations were randomized across individuals (see methods in manuscript for details). Motor performance was measured using a specialized keyboard using Matlab Psychophysics Toolbox version 3. Sleep was recorded using a digital sleep recorder (V-Amp, Brain Products, Gilching, Germany; bandwidth: DC to Nyquist frequency); Brain images were collected with a Philips Achieva 3.0T MRI system equipped with a 32-channel head coil. Participants were blind to the study hypothesis during data collection.
Timing	Mars 2021 - May 2021
Data exclusions	In total, 34 (age range 18 – 30 yo) participants completed the study. Two participants were excluded for experimental error (i.e., one participant because they were erroneously enrolled despite being left handed and the other one because of technical errors with the scanner) and one for excessive movement in the scanner (see MRI section below). The remaining 31 participants were included in closed-loop stimulation analyses (i.e., SO detection accuracy and stimulation phase, N = 31), but only 27 participants presented a complete dataset (behavior, sleep EEG and MRI data). In three participants, only the sleep EEG data was analyzed as behavioral and MRI data of the post-night session was corrupted due to experimental error (sleep EEG analyses, N = 30). For one participant, only MRI and behavioral data were analyzed due to an EEG recording default (Behavioral and MRI analyses, N = 28). Also note that for five participants, the pre-night Psychomotor Vigilance Task data was overwritten due to experimental error whereas for one participant both the pre- and post-night data were overwritten. Participant's characteristics are reported in Table S5 in the supplemental information.
Non-participation	No participant dropped-out
Randomization	Participants were not allocated into different experimental groups

Reporting for specific materials, systems and methods

We require information from authors about some types of materials, experimental systems and methods used in many studies. Here, indicate whether each material, system or method listed is relevant to your study. If you are not sure if a list item applies to your research, read the appropriate section before selecting a response.

Materials & experimental systems

n/a	Involved in the study
☒	☐ Antibodies
☒	☐ Eukaryotic cell lines
☒	☐ Palaeontology and archaeology
☒	☐ Animals and other organisms
☒	☐ Clinical data
☒	☐ Dual use research of concern
☒	☐ Plants

Methods

n/a	Involved in the study
☒	☐ ChIP-seq
☒	☐ Flow cytometry
☐	☒ MRI-based neuroimaging

Plants

Seed stocks

Report on the source of all seed stocks or other plant material used. If applicable, state the seed stock centre and catalogue number. If plant specimens were collected from the field, describe the collection location, date and sampling procedures.

Novel plant genotypes

Describe the methods by which all novel plant genotypes were produced. This includes those generated by transgenic approaches, gene editing, chemical/radiation-based mutagenesis and hybridization. For transgenic lines, describe the transformation method, the number of independent lines analyzed and the generation upon which experiments were performed. For gene-edited lines, describe the editor used, the endogenous sequence targeted for editing, the targeting guide RNA sequence (if applicable) and how the editor was applied.

Authentication

Describe any authentication procedures for each seed stock used or novel genotype generated. Describe any experiments used to assess the effect of a mutation and, where applicable, how potential secondary effects (e.g. second site T-DNA insertions, mosiacism, off-target gene editing) were examined.

Magnetic resonance imaging

Experimental design

Design type

Task-related functional MRI: block design

Design specifications

The pre-night training session consisted of 63 practice blocks (21 blocks per sequence) immediately followed by a post-training test of 9 practice blocks (3 blocks per sequence). The post-night session consisted of 63 practice blocks (21 blocks per sequence). In each block, participants practice the 5-element sequence 4 times (20 key presses). Practice blocks were alternated with 10 s rest-intervals. The 10-second rest blocks occurring between each block of motor practice served as the baseline condition modeled implicitly in the block design.

Behavioral performance measures

For each block of practice, motor performance on both the random and sequential SRTT was measured in terms of speed (median response time on correct trials, in ms) and accuracy (% of correct responses, with a trial classified as "correct" if the key pressed by the participants matches the visual cue). Performance was analysed using a Block by Condition repeated measures ANOVA. In case of violation of the sphericity assumption, Greenhouse-Geisser corrections were applied. Offline changes in performance between pre- and post-night sessions were compared between conditions using a one-way repeated measure ANOVA with condition as within-subject factor.

Acquisition

Imaging type(s)

functional and structural

Field strength

3T

Sequence & imaging parameters

1. Task-related acquired using an ascending gradient EPI pulse sequence for T2*-weighted images (TR = 2000 ms; TE = 29.8 ms; multiband factor 2; flip angle = 90°; 54 transverse slices; slice thickness = 2.5 mm; interslice gap = 0.2 mm; voxel size = 2.5 × 2.5 × 2.5 mm3; field of view = 210 × 210 × 145.6 mm3; matrix = 84 × 82) for each participant (max. 1200 dynamic scans).
2. Resting-state fMRI data were also collected prior and immediately after the training and overnight retest sessions with the same EPI sequence as above (data not reported here).
3. Field maps (TR = 1500 ms; TE = 3.5 ms; flip angle = 90°; 42 transverse slices; slice thickness = 3.75 mm; interslice gap = 0 mm; voxel size = 3.75 × 3.75 × 3.75 mm3; field of view = 240 × 240 × 157.5 mm3; matrix = 64 × 64)
4. Three sets of EPI images using reversed phase-encoding polarity (TR = 2000 ms; TE = 29.8 ms; multiband factor 2; flip angle = 90°; 54 transverse slices; slice thickness = 2.5 mm; interslice gap = 0.2 mm; voxel size = 2.5 × 2.5 × 2.5 mm3; field of view = 210 × 210 × 145.6 mm3; matrix = 84 × 82, 6 dynamic scans).
5. High-resolution T1-weighted structural images were acquired with a MPRAGE sequence (TR = 9.5 ms, TE = 4.6 ms, TI = 858.1 ms, FA = 9°, 160 slices, FoV = 250 × 250 mm2, matrix size = 256 × 256 × 160, voxel size = 0.98 × 0.98 × 1.20 mm3)

Area of acquisition

Whole brain

Diffusion MRI ☐ Used ☒ Not used

Preprocessing

Preprocessing software

Functional images were preprocessed and analyzed using SPM12 implemented in Matlab (VERSION). Preprocessing included the realignment of the functional time series, segmentation of the structural T1-image, coregistration of functional images to the structural T1-image, spatial normalization (see below) and spatial smoothing (Gaussian kernel, 8 mm full-width at half-maximum [FWHM]).

Normalization

Spatial normalization was performed on both functional and anatomical images using non-linear registration with the MNI template in SPM12 that makes use of individual flow fields.

Normalization template

MNI template

Noise and artifact removal

Movement parameters derived from the realignment step were entered as regressors of no interest in first-level fixed effects

GLM (see below)

Volume censoring

No censoring was applied

Statistical modeling & inference

Model type and settings

We used a mass-univariate approach for the analysis of the fMRI data. The analysis was conducted in 2 serial steps accounting for fixed and random effects, respectively. At the first level, changes in brain responses were estimated using a general linear model including the responses to task practice and their linear modulation by performance speed during task practice. The 10-second rest blocks occurring between each block of motor practice served as the baseline condition modelled implicitly in the block design. Regressors of interest consisted of box cars convolved with the canonical hemodynamic response function. Movement parameters (derived from realignment of the functional volumes) were entered as regressors of no interest. High-pass filtering with a cut-off period of 128 s served to remove low-frequency drifts from the time series and an autoregressive (order 1) plus white noise model and a restricted maximum likelihood (ReML) algorithm was used to estimate serial correlations in fMRI signal. The second level analyses were performed using one-sample t-tests.

Task-related functional connectivity was examined using psychophysiological interaction (PPI) analyses with a data-driven seed selection approach. For each individual, the first eigenvariate of the signal was extracted using Singular Value Decomposition of the time series across the voxels included in a 10 mm-radius sphere centered on these coordinates. Linear models were generated, at the individual level, with a first regressor representing the practice of the motor sequence (pre- and post-night sessions in each of the three reactivation conditions), a second regressor corresponding to the BOLD signal in the seed and a third regressor representing the interaction between the first (psychological) and second (physiological) regressors. To build this regressor, the underlying neuronal activity was first estimated by a parametric empirical Bayes formulation, combined with the psychological factor, and subsequently convolved with the hemodynamic response function. The individual linear contrasts testing for the interaction between the psychological and physiological regressors within and between the different runs mentioned above were then further spatially smoothed (Gaussian kernel 6 mm FWHM). The resulting contrast images were entered in a second level analysis for statistical inference at the group level (one sample t-tests), corresponding to a random effects model accounting for inter-subject variance.

Regression analyses were performed between the individuals' brain maps showing between session changes in activity/ connectivity within and between each condition and the individuals' TMR index. These regressions were performed in a second level analysis for statistical interference at the group level (one sample t-test), corresponding to a random effects model accounting for inter-subject variance. Finally, we performed exploratory regression analyses between the individuals' brain maps showing between session changes in activity/connectivity within each condition and the EEG sigma power as well as the peak amplitude of the SOs. These regression analyses were performed separately for each sleep feature (i.e., SO amplitude and sigma power) in a second level analysis for statistical interference at the group level (one sample t-test), corresponding to a random effects model accounting for inter-subject variance.

Effect(s) tested

Effect tested were: (1) main effect of task practice across conditions; (2) differences in brain activity between sessions within and between conditions, (3) regression between overnight changes in activity (within and between conditions) and covariate of interest (behavioral index and sleep indices). The same effects were tested on the connectivity maps.

Specify type of analysis: ☐ Whole brain ☒ ROI-based ☐ Both

Anatomical location(s)

The statistical analyses were performed across all the voxels of a large mask including a set of task-relevant brain regions involved in motor sequence learning processes and consisting of the primary motor cortex (M1), the supplementary motor cortex (SMA), the premotor cortex (PMC), the anterior part of the superior parietal lobule (aSPL), the hippocampus, the putamen and the caudate nucleus. These brain areas were defined with the brainnetome atlas. The probability maps for the cortical areas were thresholded at 50% for binarization while the probability maps of the subcortical areas were thresholded at 5% for binarization.

Statistic type for inference

(See Eklund et al. 2016)

Voxel-wise

Correction

Statistical inferences were performed at a threshold of $p < 0.05$ after family-wise error (FWE) correction for multiple comparisons over small volumes (SVC, 10 mm radius) located in the structures of interest reported by published work.

Models & analysis

n/a	Involved in the study
☐	☒ Functional and/or effective connectivity
☒	☐ Graph analysis
☒	☐ Multivariate modeling or predictive analysis

Functional and/or effective connectivity

The connectivity-based analyses were performed using psychophysiological interaction (PPI) analyses with a data-driven seed selection approach. For each individual, the first eigenvariate of the signal was extracted using Singular Value Decomposition of the time series across the voxels included in a 10 mm-radius sphere centered on these coordinates. Linear models were generated, at the individual level, with a first regressor

representing the practice of the motor sequence (pre- and post-night sessions in each of the three reactivation conditions), a second regressor corresponding to the BOLD signal in the seed and a third regressor representing the interaction between the first (psychological) and second (physiological) regressors. To build this regressor, the underlying neuronal activity was first estimated by a parametric empirical Bayes formulation, combined with the psychological factor, and subsequently convolved with the hemodynamic response function. The individual linear contrasts testing for the interaction between the psychological and physiological regressors within and between the different runs mentioned above were then further spatially smoothed (Gaussian kernel 6 mm FWHM). The resulting contrast images were entered in a second level analysis for statistical inference at the group level (one sample t-tests), corresponding to a random effects model accounting for inter-subject variance.
